# Network Pharmacological Analysis and Experimental Study of the Antipharyngitis Mechanism of the Chaiqin Qingning Capsule

**DOI:** 10.1155/2022/5616942

**Published:** 2022-04-28

**Authors:** Can Wang, Hongjin Gao, Lianzhan Huang, Zhen Wang, Xuansheng Ding

**Affiliations:** Department of Clinical Pharmacy, School of Basic Medicine and Clinical Pharmacy, China Pharmaceutical University, Nanjing 211198, China

## Abstract

**Objective:**

The study aimed to explore the active composition and mechanism of the Chaiqin Qingning capsule (CQQN) against pharyngitis based on the network pharmacology and through using a pharyngitis rat model.

**Methods:**

The active ingredients and targets of CQQN were queried using the TCMSP database. Disease-related target genes were queried in the DrugBank, GeneCards, OMIM, and DisGeNEt databases using “pharyngitis” as the search term. The STRING database was used to establish a protein-protein interaction (PPI) network. GO function enrichment and KEGG pathway enrichment analyses were performed to identify active components and key targets. Cytoscape software (version 3.7.2) was used to construct an active component/target gene/enrichment pathway network. AutoDock software was used to select the best binding target for molecular docking. The effect of CQQN was verified in the pharyngitis rats.

**Results:**

Network pharmacology showed 30 active compounds in CQQN with 240 targets, including 54 for the treatment of pharyngitis. Potential active ingredients included quercetin, kaempferol, stigmasterol, saikosaponin D, and isorhamnetin. The key targets involved were AKT1, TNF, IL-6, and IL-1*β*. Signaling pathways included virus infection, TNF, IL-17, and cancer pathways. The molecular docking results showed that the critical components in CQQN had good potential for binding to key target genes. Animal experiments showed that CQQN could significantly reduce the expression of TNF-*α*, IL-1*β*, IL-6, and IL-17 in the serum of rats with pharyngitis (*P* < 0.05). Hematoxylin and eosin staining showed that the inflammatory state of pharyngeal tissue in rats was significantly reduced compared to that in the model group.

**Conclusion:**

CQQN can improve pharyngitis by regulating the TNF and IL-17 signaling pathways. The study makes a positive exploration and provides a new idea for a more comprehensive and in-depth excavation of CQQN with an intervention effect on pharyngitis and other upper respiratory diseases in the future.

## 1. Introduction

Pharyngitis is inflammation of pharyngeal mucosa and surrounding lymphoid tissue, characterized by congestion and edema [1]. The main *symptom* of *pharyngitis* is a sore, dry, or itchy throat. Pharyngitis is usually caused by various viral or bacterial infections. The virus is the most common cause, accounting for about 40%-60% of cases. The second cause is a bacterial infection, accounting for about one-third of children's pharyngitis, but only about 5%-10% of that in adults [2, 3]. Signs and symptoms of pharyngitis caused by bacteria, viruses, and other microorganisms usually overlap and are difficult to distinguish. This also creates difficulties in the proper use of drugs in practice. Anti-infection treatments (penicillins, erythromycins, and sulfonamides) are the first choice for acute bacterial pharyngitis. Although there is a specific therapeutic effect, long-term drug use significantly increases bacterial resistance, resulting in a poor therapeutic effect and affecting patients' adherence [4].

Chaiqin Qingning capsule (CQQN) is a Chinese patent medicine preparation composed of Radix Bupleuri, Baicalin, and Artificial Calculus Bovis. Radix Bupleuri has antipyretic, anti-inflammatory, immune enhancement, antibacterial, and antitumor pharmacological effects. Baicalin has antipyretic, antibacterial, anti-inflammatory, antiviral, antiendotoxin, antioxidant, and antitumor effects. Artificial Calculus Bovis has the pharmacological effects of clearing away heat, detoxifying, and regulating immunity [5]. CQQN is clinically used for the treatment of acute pharyngitis and sore throat. It has the efficacy of shortening the course of the disease, improving clinical symptoms and signs such as coughing, nasal obstruction, runny nose, and pharyngeal redness with high safety and effectiveness, and it has no apparent adverse reactions [6, 7]. In several multicenter randomized controlled trials, the clinical efficacy evaluation results of CQQN showed that it could effectively relieve fever and sore throat caused by pharyngitis [8]. However, the pharmacological mechanism of CQQN is unclear [9].

The clinical effect of traditional Chinese medicine (TCM) results from the comprehensive action between its active component group and the complex biological network of the body. Integration analysis between the active component group and the target group of the body is a challenge for studying the pharmacological mechanism of TCM [10, 11]. Through the theory and method of integrated system biology and bioinformatics, network pharmacology has carried out an overall analysis of the interaction between drugs and the body at the macro-level, bringing new research ideas to clarify the pharmacological mechanism of TCM [12, 13]. The potential active components of drugs and their possible targets can be expressed as a nested topological interaction network. By analyzing the topological properties of the network, influential molecular groups, target groups, and disease-related signaling pathways of drugs can be analyzed as a whole. Through the continuous development of predecessors, network pharmacology has been widely used to study the pharmacological mechanism of TCM and its compound, providing a rapid and effective overall analysis for pharmacological research of TCM [14, 15].

This study aimed to explore the intervention mechanism of CQQN in pharyngitis by network pharmacology and to verify the mechanism in a rat pharyngitis model. The results can provide references for follow-up research.

## 2. Materials and Methods

### 2.1. Reagents and Animals

CQQN capsules were purchased from Yangzijiang Pharmaceutical Group Co., Ltd. (China), batch number 21022312. Ammonia water was purchased from Nanjing Chemical Reagent Co., Ltd. (China), batch number 200303196X. The enzyme-linked immunosorbent assay (ELISA) kit was purchased from Jiangsu Enzyme Immunoassay Industry Co., Ltd. (China), batch number 202106.

The rats were purchased from Hangzhou Medical College, animal license number SCXK (Zhejiang) 2019-0002 and certificate number 20210628Aazz0100000604. The rats were raised under controlled humidity (55-65%), temperature (22 ± 2°C), and daily light intensity (12-h/12-h light/dark cycles). The rats had free access to water and standard diets. The experimental protocols were approved by the Committee on Laboratory Animal Care of China Pharmaceutical University, and all animals were treated with humane care according to the National Institutes of Health (USA) guidelines.

### 2.2. Database and Analysis Platform/Software

TCMSP platform (http://lsp.nwu.edu.cn/tcmsp.php/), SwissTargetPrediction (http://www.swisstargetprediction.ch/), DrugBank database (https://go.drugbank.com/), GeneCards database (https://www.genecards.org/), OMIM database (https://www.omim.org/), DisGent database (https://www.disgenet.org/home/), UniProt database (http://www.uniprot.org/), PubChem database (http://pubchem.ncbi.nlm.nih.gov/), STRING database (https://string-db.org/), protein structure database (http://www.rscb.org/), Cytoscape (version3.7.2), ChemBioDraw (version 14.0), Venny plot production platform (http://bioinformatics.psb.ugent.be/webtools/Venn/), PyMOL software, AutoDockTools 1.5.6 software, and R 4.1.0 software were used.

### 2.3. Screening of Active Compounds and Target Genes

The components of CQQN “Radix Bupleuri,” “Baicalin,” and “Artificial Calculus Bovis” were used as search terms to screen active compounds from the TCMSP and the TCM system pharmacology databases. Oral bioavailability (OB) ≥30% and drug-likeness (DL) ≥0.18 were used as screening criteria for active compounds, and related target genes were collected [16]. The standardized targets were obtained from the UniProt database.

### 2.4. Screening of Targets Related to Pharyngitis

The relevant pharyngitis targets were queried in the DisGeNET, GeneCards, OMIM, and the DrugBank databases, and duplicate targets were deleted after collection. The remaining targets were used as pharyngitis disease targets.

### 2.5. Screening Core Targets

The potential targets of CQQN on pharyngitis were obtained by matching the target genes of the active ingredients with the target genes related to pharyngitis. The Venny map was used to draw the intersection map of the active drug component and disease targets.

### 2.6. Construction of the Protein-Protein Interaction Network

The protein-protein interaction (PPI) network between targets was created by selecting “Multiple proteins” in the STRING database interface, copying the intersection targets into “List of Names,” and selecting “Homo sapiens” in “Organism.” Other parameters remain as default. The obtained PPI network was imported into the Cytoscape software and visualized using the network analyzer function.

### 2.7. GO and KEGG Enrichment Analysis

R packages including “colorspace” and “string” were installed. A Bioconductor package (http://org.Hs.eg.db) that includes “DOSE,” “clusterProfiler,” and “pathview” was used to perform the Gene Ontology (GO) biological processes and Kyoto Encyclopedia of Genes and Genomes (KEGG) pathway analysis on the common target of CQQN and pharyngitis. According to *P* < 0.05, the top ten biological processes (BP), molecular functions (MF), cellular components (CC), and the top 20 KEGG pathways were selected. Subsequently, experimental data such as active ingredients, targets, and the top 20 KEGG pathways were imported into the Cytoscape software to construct a network of “active ingredient/target/enrichment pathways.”

### 2.8. Molecular Docking Verification

The Mol2 format file of the active components of the drug was downloaded from the PubChem database, and the 3D structure of the target protein was downloaded from the PDB database. PyMOL software was used to remove water and phosphate from the protein. Then AutoDockTools and AutoDock Vina software were used for molecular docking. The binding free energy was used as the evaluation criterion for the binding degree of compounds.

### 2.9. Acute Pharyngitis Induced by Ammonia in Rats

Ten of the 50 healthy SD rats were randomly selected as the blank group. The rest were sprayed with 15% ammonia water on the throat with a throat sprayer, with a daily schedule of one spray in the morning and one at noon, 0.2-0.4 mL each spray for three days. The pharyngeal mucosa was swollen due to acute stimulation, thus forming an acute pharyngitis model [17].

The rats in the pharyngitis model were randomly divided into the model groups, which were the small-dose (138.83 mg/kg, equal to half of the clinical equivalent CQQN dose), the medium-dose (277.65 mg/kg, equal to the clinical equivalent CQQN dose), and the large-dose (555.30 mg/kg, equal to twice the clinical CQQN equivalent dose) CQQN groups. Each CQQN group was administered CQQN once a day for five days. The blank and model groups received an equal volume of 0.9% sodium chloride (NS) solution. After the last administration of CQQN or 0.9% NS, the pharyngeal mucosa of the animals was observed for quantitative rating evaluation. The pharyngeal scoring standard was based on the color of the pharynx tissue, secretion, degree of hyperemia, and swelling. The following four grades were assigned: grade (–): the rat's pharyngeal tissue was light red, moist, smooth with no congestion; grade (+): the rat's pharyngeal tissue was close to grade (-), but there were visible mild chronic congestion, poor mucosal gloss, and pharyngeal secretions; grade (++): the pharyngeal tissue of rats showed chronic congestion, dark red, and mild swelling with a small amount of secretion; and grade (+++): the pharyngeal tissue was in a state of chronic hyperemia, dark red, increased mucus secretion, and noticeable swelling [18].

### 2.10. The Expression of Inflammatory Cytokines in Serum Determined by ELISA

Upon the completion of CQQN or 0.9% NS administration, the serum of rats was taken, and the blood samples were placed at 4°C for 30 min. Serum was separated by 3000 rpm centrifugation for 10 min. Serum levels of IL-1*β*, IL-6, TNF-*α*, and IL-17 were measured by ELISA according to the kit instructions.

### 2.11. Observation of Pharyngeal Tissue Lesions

Pharyngeal mucosa and submucosa were extracted and fixed with 10% formalin solution. After hematoxylin and eosin (HE) staining, the histomorphological changes were observed under a light microscope.

### 2.12. Statistical Analysis

Excel was used for data entry, SPSS 25.0 software for data analysis, and GraphPad Prism 9.0 was used for mapping. Measurement data were expressed as mean ± standard deviation (mean ± SD). One-way analysis of variance was used for pairwise comparison between groups. *P* < 0.05 was considered statistically significant, and *P* < 0.01 indicated that the difference was extremely significant.

## 3. Results

### 3.1. Screening of Active Drug Ingredients and Target Genes

A total of 374 ingredients were obtained from the TCMSP database (353 in Radix Bupleuri, 19 in Artificial Calculus Bovis, and 2 in Baicalin). The active compounds were then screened with DL ≥0.18 and OB ≥30% as the condition. Saikosaponins A, B, C, and D were reported to be the main active components of Radix Bupleuri [19]. Finally, 30 active components of CQQN were obtained, including 21 in Radix Bupleuri, 2 in Baicalin, and 7 in Artificial Calculus Bovis (Table 1). Active components were searched, screened, and standardized by the UniProt database and SwissTargetPrediction. There were 375 Radix Bupleuri, 3 Baicalin, and 46 Artificial Calculus Bovis (Supplementary Table 1). A total of 226 drug targets were obtained after removing duplicate targets.

### 3.2. Acquisition and Screening of Targets for Pharyngitis

With the keywords “pharyngitis,” 46 pharyngitis-related targets were found in the DrugBank database, 536 in the GeneCards database, 38 in the OMIM database, and 30 in the DisGent database. After removing duplicate targets, 618 pharyngitis targets were obtained (Supplementary Table 2).

**Figure 1:**
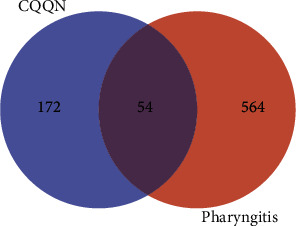
Venn diagram of intersection targets between CQQN components and pharyngitis.

The 54 common targets of the CQQN component and pharyngitis were considered potential key targets (Table 2). Targets were imported into the Venny mapping software, and the Wayne diagram was drawn, as shown in Figure 1.

### 3.3. GO and KEGG Analysis

**Figure 2:**
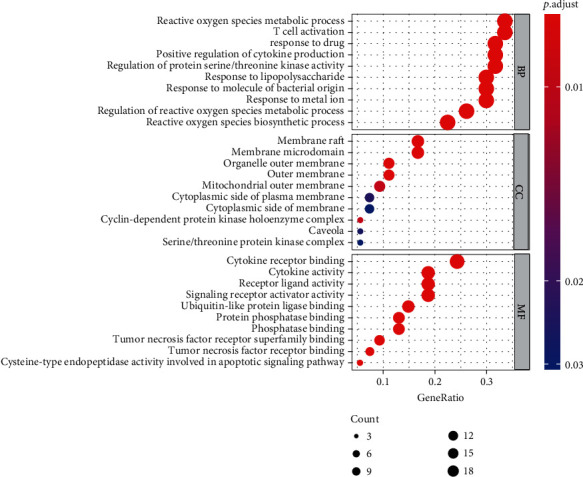
The GO enrichment analysis of CQQN targets in treating pharyngitis. Note: The *x*-axis is the enrichment gene ratio, and the *y*-axis is the biological process, cell components, and molecular function. The size represents the number of genes involved in GO enrichment, and the color represents the enrichment significance based on the corrected *P*-value.

The Bioconductor package in R software was used to perform the GO enrichment and KEGG pathway analyses on common targets of CQQN and pharyngitis. A total of 1,590 GO items were obtained (*P* < 0.05), including 1,506 BP items, 14 CC items, and 70 MF items. BP is mainly related to the response to reactive oxygen species (ROS) in the metabolic process, the response to drugs, the regulation of ROS, and the T cell activation. CC is related to the membrane raft, the side of the membrane, and the outer membrane. MF is primarily related to binding cytokines, protein phosphatase, kinase, and tumor necrosis factor (TNF) receptor superfamily (Supplementary Table 3). According to the enrichment results, the top ten BP, CC, and MF items were selected for the bubble graph, as shown in Figure 2.

**Figure 3:**
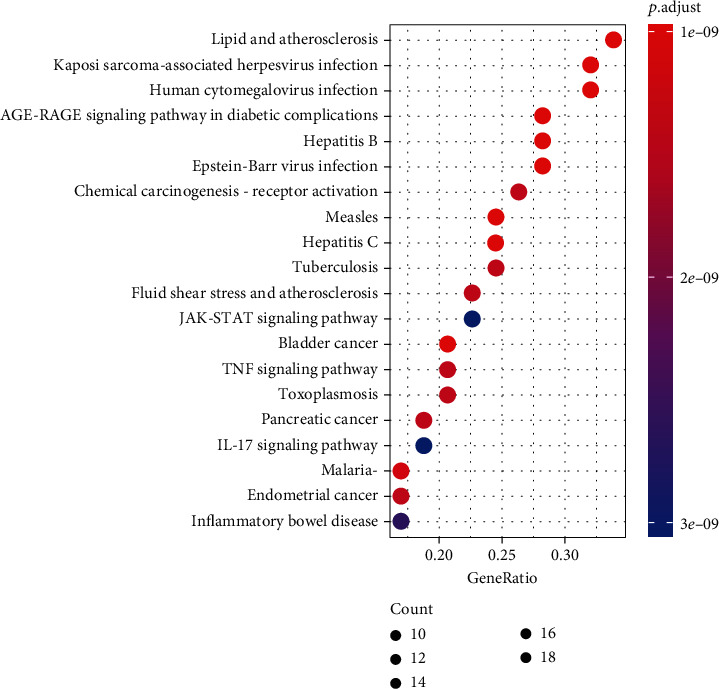
The KEGG pathway enrichment. Note: The *x*-axis is the enrichment gene count. The *y*-axis is the KEGG pathway, and the color represents the enrichment significance based on the corrected *P* value.

The KEGG enrichment analysis resulted in 139 pathways (*P* < 0.05). KEGG analysis showed that the relevant CQQN signaling pathways in the treatment of pharyngitis included virus infection, TNF, IL-17, and cancer pathways (Supplementary Table 4). The top 20 pathways with the highest number of genes for the treatment of pharyngitis were selected for the bubble chart, as shown in Figure 3. The significantly enriched genes were AKT1, IL-6, TNF, STAT3, TP53, PTGS2, EGFR, CCND1, CASP3, BCL2, STAT1, MMP9, and ICAM1.

### 3.4. Construction of Active Ingredient/Target/Enrichment Pathway Network

**Figure 4:**
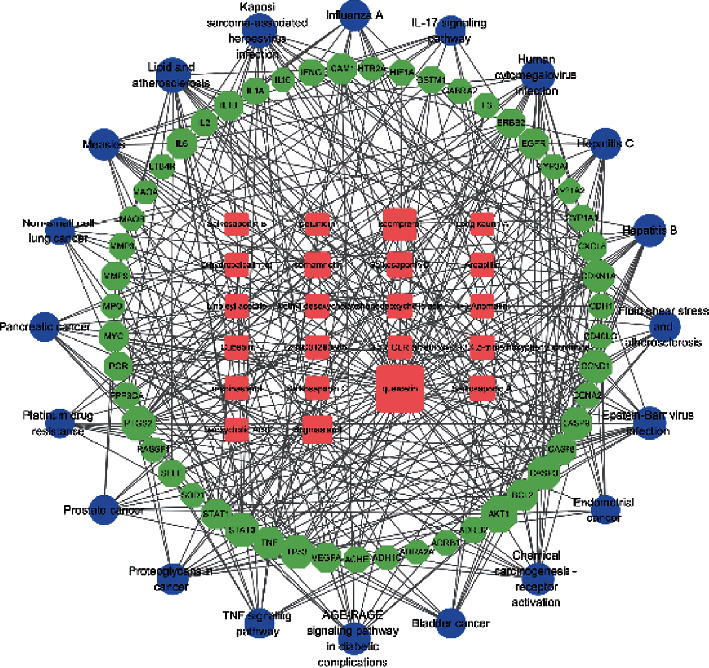
The network diagram of active components/target/enrichment pathways. Note: The square pink node represents the circular active ingredient, the green node represents the target gene, and the blue circle node represents the pathway. The size is proportional to their degree.

The active ingredients of CQQN, the potential targets for treating pharyngitis, and the top 20 KEGG-enriched pathways (*P* ≤ 0.05) were imported into the Cytoscape software to construct a network diagram of active components/target/enrichment pathways. There were 93 nodes (22 compounds, 51 targets, and 20 pathways) and 347 interacting edges in the network, as shown in Figure 4. Each compound corresponds to multiple targets in the network diagram, indicating that multiple targets may synergistically treat pharyngitis with CQQN. The topological analysis of the results was carried out using Cytoscape software, as shown in Figure 4. The higher the degree, the higher the number of targets related to this ingredient and pathway, and the greater the research significance [20].

Among the top ten components with a higher degree, there were four flavonoids (quercetin, kaempferol, isorhamnetin, and 3,5,6,7-tetramethoxy-2 - (3,4,5-trimethoxyphenyl) chromone), three saponins (saikosaponin D, saikosaponin A, and saikosaponin B), one sterol component (stigmasterol), one lignan component (cubebin), and a steroid compound (chenodeoxycholic acid). The results show that these compounds may be the key components of CQQN in the treatment of pharyngitis, as shown in Table 3.

### 3.5. PPI Network Construction

**Figure 5:**
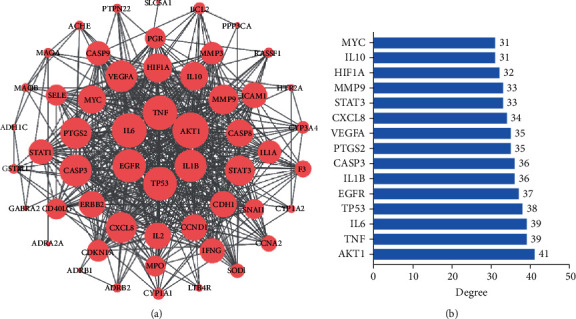
(a) The protein-protein interaction (PPI) network. (b) Top 15 targets of degree in the PPI network. Note: the nodes represent targets, and the size shows their degree in the network.

The intersection target of components and diseases was imported into the STRING database for analysis to obtain the PPI network. The species were restricted to “Homo sapiens” and filtered with confidence ≥0.4. The results were imported into the Cytoscape software for visual analysis. The larger the circle label, the higher the degree, the more associated targets, and the greater the importance of the research. A total of 54 nodes and 566 interaction lines were obtained (Figure 5(a)). After calculation, the average degree of the freedom of nodes in PPI was 21. The top 15 targets in the PPI network were selected, which were AKT1, TNF, IL-6, TP53, EGFR, IL-1*β*, CASP3, PTGS2, VEGFA, CXCL8 STAT3, MMP9, HIF1A, IL10, and MYC (Figure 5(b)). These targets are suggested to be the potential key targets of CQQN in treating pharyngitis.

### 3.6. Molecular Docking Analysis

The molecular docking technology was used to predict the binding ability of the active ingredients of CQQN with potential targets. The top ten active ingredients with higher degrees obtained by the topological analysis were used as ligands, and the top 15 key targets with higher degrees selected from the results of the PPI network and the KEGG analysis were receptors for the prediction of binding ability. The docking result retained only the highest absolute value of predicted binding affinity (kcal/mol) for each pair of molecular docking. It is generally believed that binding energy of less than -4.25 kcal/mol indicates that the ligand has a specific binding activity to the receptor, less than -5.0 kcal/mol has better binding activity, and less than -7.0 kcal/mol has strong binding activity.

**Figure 6:**
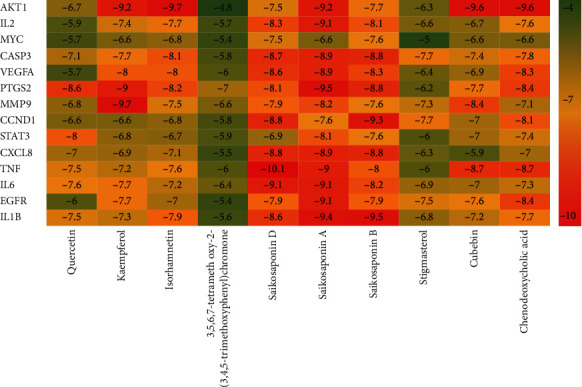
Heat maps of molecular docking scores of the active ingredients of the target CQQN protein in the treatment of pharyngitis.

**Figure 7:**
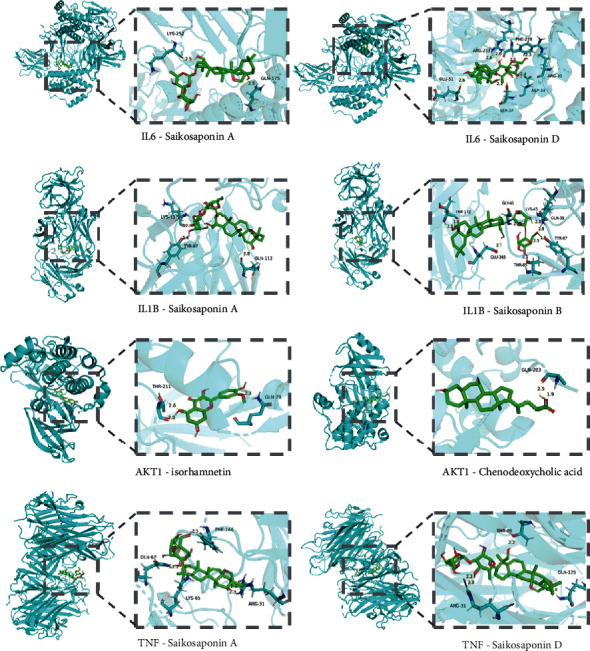
Molecular docking patterns of the active ingredients of the target CQQN protein in the treatment of pharyngitis.

The ten active pharmaceutical ingredients were bound to 14 target proteins (TP50 was deleted because the ligand structure could not be found), and the data obtained were analyzed by a heat map, as shown in Figure 6. Among them, the ingredients with a docking score ≤ -9.0 kcal/mol accounted for 12.1% (17/140); the ingredients with a docking score between -9.0 and -7.0 kcal/mol accounted for 51.4% (72/140), and the ingredients with a docking score ≥ -7.0 kcal/mol accounted for 36.4% (51/140). The results show that the active ingredients of CQQN have strong binding ability with key targets. Five typical components (saikosaponin D, isorhamnetin, chenodeoxycholic acid, saikosaponin A, and saikosaponin B) and four key targets (TNF, IL-6, IL-1*β*, and AKT1) were selected for the analysis of the binding mode. As shown in Figure 7, the binding mode is mainly hydrogen bonding, and the hydrophobic interaction further stabilizes the compound-protein structure.

### 3.7. Effect of CQQN on the Rat Model of Acute Pharyngitis

#### 3.7.1. Observation of the General State of an Acute Pharyngitis Rat Model

All rats in the blank group were in good physical condition, had bright fur color, and had normal eating and drinking patterns. After modeling, all rats had swelling and congestion of the pharynx, and oral secretions gradually increased. Some rats had local ulceration of the mouth and lips, and superficial ulcers were formed in the pharynx. After continuous administration of CQQN for five days, the redness and swelling of the small-, medium-, and large-dose groups of rats in the CQQN were reduced to varying degrees. The amount of secretion was significantly reduced compared to that of the model group (Table 4).

#### 3.7.2. Effect on Pathological Changes in Pharynx Tissue in Rats with Acute Pharyngitis

**Figure 8:**
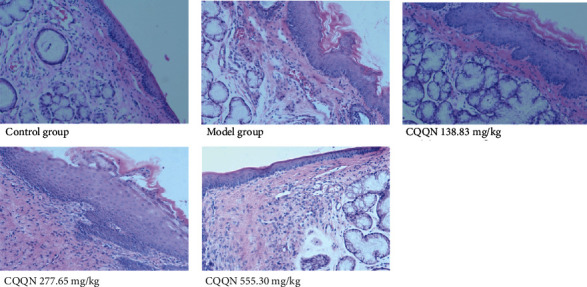
Results of histopathological HE staining results of pharynx of rats in each group (HE, ×200).

In the structure of the blank group, the rat pharynx tissue and the submucosal glands were normal, and there were no inflammatory cells. In the structure of the model group, the rat pharynx tissue changed with the congested mucosal layer of blood vessels. The submucosal layer of connective tissue proliferated, and many inflammatory cells infiltrated. The pharyngeal mucosa of rats in the high-dose CQQN group was basically restored to normal, and the submucosal inflammatory cells and mucosal glands were significantly reduced. In the medium-dose CQQN group, a small number of inflammatory cells were observed in the mucosa and submucosa in the pharyngeal tissue. In the small-dose CQQN group, the pharyngeal mucosa of rats became thinner. Inflammatory cells were significantly reduced, and the submucosal mucosal glands were basically normal, as shown in Figure 8.

The histopathological grade of the pharynx in the model group was significantly higher than that of the blank group (*P* < 0.01). Compared to the model group, the pharyngeal tissues of the CQQN small-, medium-, and large-dose groups improved significantly (*P* < 0.01), as shown in Table 5.

#### 3.7.3. Serum Levels of IL-6, IL-1*β*, TNF-*α*, and IL-17 in Rats of Each Group

**Figure 9:**
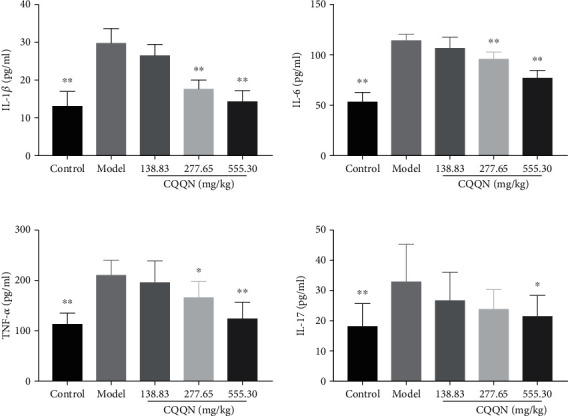
Serum levels of IL-6, IL-1*β*, TNF-*α*, and IL-17 in each group of rats. Note: ^∗^*P* < 0.05 and ^∗∗^*P* < 0.01 vs. model group, $\bar{x}\pm \text{s}$, *n* = 10.

Compared to the blank group, the levels of TNF-*α*, IL-1*β*, IL-6, and IL-17 in the model group increased significantly (*P* < 0.01). Compared to the model group, the CQQN large-dose group significantly reduced the levels of IL-6, TNF-*α*, and IL-1*β* (*P* < 0.01) and significantly reduced the level of IL-17 (*P* < 0.05). The medium-dose CQQN group significantly reduced the levels of IL-1*β* and IL-6 (*P* < 0.01) and significantly reduced the TNF-*α* level (*P* < 0.05), as shown in Table 6 and Figure 9.

## 4. Discussion

Pharyngitis is an inflammation of the upper respiratory tract. Sore throat and dysphagia in the disease process will seriously affect patients' daily lives and even cause serious complications, such as rheumatic heart disease, glomerulonephritis after streptococcal infection, sepsis, or autoimmune diseases [22]. CQQN is a TCM preparation containing complex ingredients and is often used clinically to treat upper respiratory tract diseases such as sore throat and pharyngitis.

Network pharmacology results show that quercetin, kaempferol, stigmasterol, saikosaponin A, saikosaponin C, saikosaponin D, isorhamnetin, and other ingredients play a vital role in the treatment of pharyngitis by CQQN. Quercetin is a flavonoid compound. It is considered the most effective ROS scavenger and inhibits the production of several proinflammatory factors, such as TNF-*α* and NO [23]. In vitro treatment of activated T cells with quercetin blocks IL-12-induced tyrosine phosphorylation of JAK2, TYK2, STAT3, and STAT4, resulting in a decrease in IL-12-induced T cell proliferation and Th1 differentiation [24]. Several in vitro studies using different cell lines have shown that quercetin inhibits lipopolysaccharide (LPS)-induced TNF-*α* production in macrophages [25, 26] and LPS-induced IL-8 production in lung A549 cells [27]. Kaempferol has anti-inflammatory, antioxidant, and immunomodulatory pharmacological properties. Kaempferol can significantly improve lung ischemia-reperfusion injury, inhibit the release of inflammatory factors including IL-6 and TNF-*α* in bronchoalveolar lavage fluid, and reduce the reaction of oxidative stress [28]. Kaempferol can regulate the phosphorylation of I*κ*B-*α* and p65 and inhibit inflammation in vitro and in vivo by regulating the MAPK and NF-*κ*B pathways. NF-*κ*B, which is involved in the immune response and the inflammatory response, is a familiar molecular target for the treatment of pharyngitis [29]. Saikosaponin is a triterpene saponin that has a variety of pharmacological activities, including anti-inflammatory and antioxidant effects. Saikosaponin can reduce inflammation and regulate autophagy by inhibiting the PI3k/Akt/mTOR signaling pathway [30]. Stigmasterol has antioxidant, anti-inflammatory, antitumor, and other pharmacological activities and can significantly suppress the expression of proinflammatory mediators (TNF-*α*, IL-6, IL-1*β*, iNOS, and COX-2) and increase the expression of anti-inflammatory cytokines (IL-10) through downregulating the expression of NF-kBp65 (inhibiting p-IKB-*α* activation) and p38MAPK [31]. Isorhamnetin has various effects such as protecting cardiovascular and cerebrovascular, antitumor, anti-inflammatory, and antiviral activities. Treatment with isorhamnetin inhibits the phosphorylation of the mitogen-activated protein kinase (MAPK) and NF-*κ*B pathways induced by TNF-*α* [32]. The above shows that the key active ingredients of CQQN all have good anti-inflammatory, antioxidant, or immunomodulatory effects.

The GO analysis result shows that the cytokine receptor binding and TNF receptor binding are at the top. It indicates that these receptors may be the primary drug targets of CQQN in the treatment of pharyngitis. TNF is a multifunctional cytokine. It plays an essential role in the pathophysiology of several diseases [33]. TNF-*α* is an inflammatory cytokine produced by macrophages/monocytes during acute inflammation and is responsible for a wide range of signaling events within cells, leading to necrosis or apoptosis [34].

The results of the KEGG pathway enrichment analysis show that the targets of CQQN are mainly concentrated in virus infection and TNF, IL-17, and cancer pathways. Studies have found that the TNF signaling pathway plays a key role in the inflammatory response. TNF-*α* is the most important cytokine in this pathway by upregulating MAPK, ERK, NF-*κ*B, and other signaling pathways to induce apoptosis and plays a proinflammatory role [35]. MAPK is related to cell proliferation and immune regulation. It can manipulate key host cell signals to induce IL-1*β*, IL-6, TNF-*α*, and other inflammatory mediators [36]. IL-17 is the signature cytokine of the Th17 cell and is secreted as a homodimer or as a heterodimer with IL-17F [37]. IL-17 is a crucial mediator of mucosal surveillance and barrier integrity. The most prominent function of IL-17 is to provide a protective inflammatory response against pathogens at boundary tissues, such as the skin, intestine, and lungs. In pharyngitis or tonsillitis caused by streptococcus, peripheral T cells can be induced to differentiate into Th17 cells. IL-17 secreted by Th17 cells has a proinflammatory effect [38]. In addition, IL-17 is involved in epithelial cell and neutrophil-mediated immune responses against extracellular microbes and autoimmune diseases' pathogenesis [39, 40]. Consequently, CQQN is likely to treat pharyngitis by participating in the IL-17 signal pathway.

The PPI topological analysis shows 16 strongly associated proteins, among which AKT1, TNF, IL-6, and IL-1*β* are the predicted targets of CQQN. The molecular docking results showed that the active components of CQQN had a strong affinity with the key targets, a stable hydrogen bond at the binding site, and a stable conformation and good binding activity. It can provide a theoretical basis for CQQN treating pharyngitis at the molecular level.

Through the animal experiment, we found that the pharyngeal mucosa of pharyngitis rats in each dose group improved to various degrees after CQQN was administered for five days. Under a light microscope, it was found that the pathological changes of the pharyngeal tissue in CQQN were less than that in the model group. Inflammatory cells in the submucosa were significantly reduced, and mucosal glands were also decreased.

IL-6, IL-1*β*, TNF-*α*, and IL-17 in the medium and large-dose CQQN groups were significantly lower than those in the model group. It is suggested that CQQN inhibits the development of inflammation by reducing the production of proinflammatory factors and mediators and blocking the transmission of proinflammatory signals and playing a role in the treatment of pharyngitis.

Saikosaponin A has been reported to reduce serum IL-6, TNF-*α*, and IL1*β* after anorectal surgery in rats [41], and quercetin can reduce levels of TNF-*α* and IL-6 in mouse nasal mucosa tissue [42]. Stigmasterol has been reported to be related to reducing critical cytokines in the pain and inflammation (TNF-*α*, IL-1*β*, and IL-6) [43]. This shows that the active ingredients in CQQN have potential therapeutic anti-inflammatory and immunomodulatory effects.

This study screened potential active ingredients, key targets, and CQQN pathways of CQQN in the treatment of pharyngitis through network pharmacology. The results show that CQQN can intervene in the expression of inflammatory factors through multiple components, targets, and pathways. The results of animal experiments indicate that CQQN exerts a therapeutic effect by downregulating proinflammatory cytokines (TNF-*α*, IL-6, IL-1*β*, and IL-17) to inhabit the TNF and IL-17 signaling pathways. However, this study only conducted a single efficacy test through the pharyngitis rat model; more studies of CQQN need to be conducted. This study provides ideas and a basis for a more comprehensive and in-depth exploration of TCM with intervention effects on pharyngitis in the future.

## Figures and Tables

**Table 1:** The main active ingredients of CQQN.

Herb	ID	Compound	OB (%)	DL
Radix Bupleuri	MOL001645	Linoleyl acetate	42.1	0.2
Radix Bupleuri	MOL002776	Baicalin	40.12	0.75
Radix Bupleuri	MOL000449	Stigmasterol	43.83	0.76
Radix Bupleuri	MOL000354	Isorhamnetin	49.6	0.31
Radix Bupleuri	MOL000422	Kaempferol	41.88	0.24
Radix Bupleuri	MOL004598	3,5,6,7-tetramethoxy-2-(3,4,5-trimethoxyphenyl)chromone	31.97	0.59
Radix Bupleuri	MOL004609	Areapillin	48.96	0.41
Radix Bupleuri	MOL013187	Cubebin	57.13	0.64
Radix Bupleuri	MOL004624	Longikaurin A	47.72	0.53
Radix Bupleuri	MOL004628	Octalupine	47.82	0.28
Radix Bupleuri	MOL004644	Sainfuran	79.91	0.23
Radix Bupleuri	MOL004648	Troxerutin	31.6	0.28
Radix Bupleuri	MOL004653	(+)-Anomalin	46.06	0.66
Radix Bupleuri	MOL004702	Saikosaponin c_qt	30.5	0.63
Radix Bupleuri	MOL004718	*α*-Spinasterol	42.98	0.76
Radix Bupleuri	MOL000490	Petunidin	30.05	0.31
Radix Bupleuri	MOL000098	Quercetin	46.43	0.28
Radix Bupleuri	MOL004635	Saikosaponin A	32.39	0.09
Radix Bupleuri	MOL004636	Saikosaponin B	6.70	0.13
Radix Bupleuri	MOL004701	Saikosaponin C	5.12	0.05
Radix Bupleuri	MOL004637	Saikosaponin D	34.39	0.09
Artificial cow-bezoar	MOL008838	Methyl (4R)-4-[(3R,5S,7S,8R,9S,10S,12S,13R,14S,17R)-3,7,12-trihydroxy-10,13-dimethyl-2,3,4,5,6,7,8,9,11,12,14,15,16,17-tetradecahydro-1H-cyclopenta[a]phenanthren-17-yl]pentanoate	32.32	0.76
Artificial cow-bezoar	MOL008839	Methyl desoxycholate	34.63	0.73
Artificial cow-bezoar	MOL008845	Deoxycholic acid	40.72	0.68
Artificial cow-bezoar	MOL008846	ZINC01280365	46.38	0.49
Artificial cow-bezoar	MOL000953	CLR	37.87	0.68
Artificial cow-bezoar	MOL008841	Taurocholate	10.25	0.87
Artificial cow-bezoar	MOL008842	Chenodeoxycholic acid	27.17	0.69
Baicalin	MOL002913	Dihydrobaicalin_qt	40.04	0.21
Baicalin	MOL002935	Baicalin	40.12	0.75

**Table 2:** The 54 potential antipharyngitis target genes of active ingredients.

UniProt ID	Protein name	Gene name
P08254	Stromelysin-1	MMP3
P27338	Amine oxidase [flavin-containing] B	MAOB
P05177	Cytochrome P450 1A2	CYP1A2
Q9Y2R2	Tyrosine-protein phosphatase non-receptor type 22	PTPN22
P09488	Glutathione S-transferase Mu 1	GSTM1
P22301	Interleukin-10	IL10
Q08209	Serine/threonine-protein phosphatase 2B catalytic subunit alpha isoform	PPP3CA
P00533	Epidermal growth factor receptor	EGFR
P00441	Superoxide dismutase [Cu-Zn]	SOD1
P21397	Amine oxidase [flavin-containing] A	MAOA
P60568	Interleukin-2	IL2
P04626	Receptor tyrosine-protein kinase erbB-2	ERBB2
P12830	Cadherin-1	CDH1
P38484	Interferon gamma	IFNG
P10415	Apoptosis regulator Bcl-2	BCL2
P08913	Alpha-2A adrenergic receptor	ADRA2A
P05362	Intercellular adhesion molecule 1	ICAM1
P04798	Cytochrome P450 1A1	CYP1A1
Q14790	Caspase-8	CASP8
Q9NWT6	Hypoxia-inducible factor 1-alpha	HIF1A
P10145	Interleukin-8	CXCL8
P16581	E-selectin	SELE
P06401	Progesterone receptor	PGR
O95863	Zinc finger protein SNAI1	SNAI1
P04637	ETS domain-containing protein Elk-1	TP53
P08588	Beta-1 adrenergic receptor	ADRB1
P01375	Tumor necrosis factor	TNF
P55211	Caspase-9	CASP9
P14778	Interleukin-1 alpha	IL1A
P38936	Cyclin-dependent kinase inhibitor 1	CDKN1A
P05164	Myeloperoxidase	MPO
P31749	RAC-alpha serine/threonine-protein kinase	AKT1
P40763	Signal transducer and activator of transcription 3	STAT3
P13866	Sodium/glucose cotransporter 1	SLC5A1
P35354	Prostaglandin G/H synthase 2	PTGS2
P00326	Alcohol dehydrogenase 1C	ADH1C
P01584	Interleukin-1 beta	IL-1*β*
P24385	G1/S-specific cyclin-D1	CCND1
P47869	Gamma-aminobutyric acid receptor subunit alpha-2	GABRA2
P13726	Tissue factor	F3
P28223	5-hydroxytryptamine 2A receptor	HTR2A
P07550	Beta-2 adrenergic receptor	ADRB2
P15692	Vascular endothelial growth factor A	VEGFA
P01106	Myc proto-oncogene protein	MYC
P20248	Cyclin-A2	CCNA2
P22303	Acetylcholinesterase	ACHE
P29965	CD40 ligand	CD40LG
P42224	Signal transducer and activator of transcription 1-alpha/beta	STAT1
Q9NS23	Ras association domain-containing protein 1	RASSF1
P08684	Cytochrome P450 3A4	CYP3A4
P05231	Interleukin-6	IL-6
Q15722	Leukotriene B4 receptor 1	LTB4R
P42574	Caspase-3	CASP3
P14780	Matrix metalloproteinase-9	MMP9

**Table 3:** The top ten active ingredients in degree value.

Ingredient	Chemical name	Degree
Flavonoid	Quercetin	38
Flavonoid	Kaempferol	16
Sterol	Stigmasterol	9
Saponin	Saikosaponin D	4
Flavonoid	Isorhamnetin	4
Steroid	Chenodeoxycholic acid	3
Saponin	Saikosaponin A	3
Saponin	Saikosaponin B	2
Lignans	Cubebin	2
Flavonoid	3,5,6,7-tetramethoxy-2-(3,4,5-trimethoxyphenyl)chromone	2

**Table 4:** Rat pharyngeal tissue scores in each group.

Group	Dose (mg/kg)	*N*	—	+	++	+++
Blank group	—	10	10	—	—	—
Model group	—	10	—	—	2	8
CQQN group	138.83	10	1	5	1	3
	277.65	10	3	5	2	—
	555.30	10	8	2	—	—

**Table 5:** Histopathological grading of pharyngeal tissue of rats in each group.

Group	Dose (mg/kg)	*n*	Pharyngeal histopathologic classification			
			-	+	++	+++
Blank group	—	10^∗∗^	10	0	0	0
Model group	—	10	0	0	0	10
CQQN group	138.83	10^∗∗^	1	6	2	1
	277.65	10^∗∗^	5	4	1	0
	555.30	10^∗∗^	8	2	0	0

Note: ^∗^*P* < 0.05 and ^∗∗^*P* < 0.01 vs. model group. “-” means that the mucosa of the pharynx and submucosa is normal. “+” indicates that the scaly epithelium of the pharynx has little hyperplasia and a small number of inflammatory cells infiltrated under the mucosa. “++” means scaly epithelium hyperplasia of the pharynx mucosa, and inflammatory cell infiltration is seen in the lower layer. “+++” means the presence of the pharynx mucosa scaly epithelium hyperplasia with a large number of inflammatory cells infiltrated in the mucosa [21].

**Table 6:** Serum levels of IL-6, IL-1*β*, TNF-*α*, and IL-17 in each group of rats ($\bar{x}\pm \text{s}$, *n* = 10).

Group	Dose (mg/kg)	IL-6	IL-1*β*	TNF-*α*	IL-17
Blank group	—	54.49 ± 9.50^∗∗^	13.24 ± 3.84^∗∗^	116.67 ± 31.72^∗∗^	18.01 ± 7.41^∗∗^
Model group	—	116.23 ± 5.58	29.89 ± 3.94	213.99 ± 29.40	33.27 ± 12.26
CQQN group	138.83	108.25 ± 9.61	26.79 ± 2.72	200.02 ± 41.13	26.71 ± 9.44
	277.65	96.91 ± 7.23^∗∗^	17.83 ± 2.32^∗∗^	170.34 ± 30.73^∗^	23.90 ± 6.53
	555.30	78.39 ± 7.68^∗∗^	14.37 ± 2.79^∗∗^	127.13 ± 32.95^∗∗^	21.66 ± 6.78^∗^

Note: ^∗^*P* < 0.05 and ^∗∗^*P* < 0.01 vs. model group.

## Data Availability

The data used to support the findings of this study are available in the supplementary information of the manuscript.

## Supplementary Materials

Supplementary Materials Supplementary Table 1: information of predicted CQQN targets. Supplementary Table 2: information of known pharyngitis-related targets. Supplementary Table 3: top 10 GO enrichment of biological processes (BP), cellular components (CC), and molecular functions (MF) with their representative enriched terms (one per cluster). Supplementary Table 4: top 20 KEGG pathway.

## References

[1] Kociolek L. K., Shulman S. T. (2012). Pharyngitis. *Annals of internal medicine*.

[2] Maya-Barrios A., Lira-Hernandez K., Jiménez-Escobar I. (2021). Limosilactobacillus reuteri ATCC PTA 5289 and DSM 17938 as adjuvants to improve evolution of pharyngitis/tonsillitis in children: randomised controlled trial. *Beneficial microbes*.

[3] Khan A., Davis D., Brown L. (2021). A comparison of diagnostic accuracy of a rapid antigen detection test in screening for group A streptococcal throat infection between 3- to 10-year-old (children and preadolescents) and 11- to 21-year-old (adolescents). *Cureus*.

[4] Di Muzio F., Barucco M., Guerriero F. (2016). Diagnosis and treatment of acute pharyngitis/tonsillitis: a preliminary observational study in general medicine. *European Review for Medical and Pharmacological Sciences*.

[5] Zhu Z., Zhao L., Liu X. (2010). Comparative pharmacokinetics of baicalin and wogonoside by liquid chromatography-mass spectrometry after oral administration of Xiaochaihu Tang and radix scutellariae extract to rats. *Journal of Chromatography B, Analytical Technologies in the Biomedical and Life Sciences*.

[6] Wang P., Zhou Z. (2020). Efficacy and safety of the Chaiqin Qingning capsule on acute upper respiratory tract infection in children. *Clinical Journal of Chinese Medicine*.

[7] Xie W., Lei Y. (2020). Clinical study on Chaiqin Qingning capsule in the treatment of air temperature disease and heat attack on lungs syndrome in the children with upper respiratory tract infection. *China Modern Doctor*.

[8] Tang W., Yao X., Xia F. (2018). Modulation of the gut microbiota in rats by Hugan Qingzhi tablets during the treatment of high-fat-diet-induced nonalcoholic fatty liver disease. *Oxidative Medicine and Cellular Longevity*.

[9] Shen X., Zhao Z., Wang H., Guo Z., Hu B., Zhang G. (2017). Elucidation of the anti-inflammatory mechanisms of Bupleuri and Scutellariae Radix using system pharmacological analyses. *Mediators of Inflammation*.

[10] Tao W., Xu X., Wang X. (2013). Network pharmacology-based prediction of the active ingredients and potential targets of Chinese herbal Radix Curcumae formula for application to cardiovascular disease. *Journal of Ethnopharmacology*.

[11] Wang N., Zhu F., Shen M. (2019). Network pharmacology-based analysis on bioactive anti-diabetic compounds in Potentilla discolor bunge. *Journal of Ethnopharmacology*.

[12] Yuan H., Ma Q., Cui H. (2017). How can synergism of traditional medicines benefit from network pharmacology?. *Molecules*.

[13] Hopkins A. L. (2008). Network pharmacology: the next paradigm in drug discovery. *Nature Chemical Biology*.

[14] Wei S., Niu M., Wang J. (2016). A network pharmacology approach to discover active compounds and action mechanisms of San-Cao Granule for treatment of liver fibrosis. *Drug Design, Development and Therapy*.

[15] Hao D. C., Xiao P. G. (2014). Network pharmacology: a Rosetta Stone for traditional Chinese medicine. *Drug Development Research*.

[16] Liu H., Wang J., Zhou W., Wang Y., Yang L. (2013). Systems approaches and polypharmacology for drug discovery from herbal medicines: an example using licorice. *Journal of Ethnopharmacology*.

[17] Gao Y., Shu A., Zhu Y. (2020). Study on the therapeutic effect of Liuling Jiedu Wan on acute pharyngitis. *Journal Nanjing University of Traditional Chinese Medicine*.

[18] Chen Y. H., Luo R., Lei S. S. (2020). Anti-inflammatory effect of Ganluyin, a Chinese classic prescription, in chronic pharyngitis rat model. *BMC complementary medicine and therapies*.

[19] Zhang H., Zhang S., Hu M. (2020). An integrative metabolomics and network pharmacology method for exploring the effect and mechanism of Radix Bupleuri and Radix Paeoniae Alba on anti-depression. *Journal of Pharmaceutical and Biomedical Analysis*.

[20] Berger S. I., Iyengar R. (2009). Network analyses in systems pharmacology. *Bioinformatics*.

[21] Liu B., Bai M., Peng M., Miao M. (2019). Anti-inflammatory effect and the effect on acute pharyngitis rats model of compound lobelia oral liquid. *Saudi Journal of Biological Sciences*.

[22] Yao X. Y., Bian Y. J., Gao Y. (2018). Clinical application evaluation and revision suggestions of clinical practice guideline on traditional Chinese medicine therapy alone or combined with antibiotics for acute pharyngitis. *Zhongguo Zhong Yao Za Zhi= Zhongguo Zhongyao Zazhi= China Journal of Chinese Materia Medica*.

[23] Kashyap D., Garg V. K., Tuli H. S. (2019). Fisetin and quercetin: promising flavonoids with chemopreventive potential. *Biomolecules*.

[24] Muthian G., Bright J. J. (2004). Quercetin, a flavonoid phytoestrogen, ameliorates experimental allergic encephalomyelitis by blocking IL-12 signaling through JAK-STAT pathway in T lymphocyte. *Journal of Clinical Immunology*.

[25] Cheng S. C., Huang W. C., Pang S. J. H., Wu Y. H., Cheng C. Y. (2019). Quercetin inhibits the production of IL-1*β*-induced inflammatory cytokines and chemokines in ARPE-19 cells via the MAPK and NF-*κ*B signaling pathways. *International Journal of Molecular Sciences*.

[26] Tang J., Diao P., Shu X., Li L., Xiong L. (2019). Quercetin and quercitrin attenuates the inflammatory response and oxidative stress in LPS-induced RAW264.7 Cells: in vitro assessment and a theoretical model. *BioMed research international*.

[27] Geraets L., Moonen H. J., Brauers K., Wouters E. F. M., Bast A., Hageman G. J. (2007). Dietary flavones and flavonoles are inhibitors of poly(ADP-ribose)polymerase-1 in pulmonary epithelial cells. *The Journal of Nutrition*.

[28] Yang C., Yang W., He Z. (2020). Kaempferol improves lung ischemia-reperfusion injury via antiinflammation and antioxidative stress regulated by SIRT1/HMGB1/NF-*κ*B axis. *Frontiers in Pharmacology*.

[29] Sun Z., Li Q., Hou R. (2019). Kaempferol-3-O-glucorhamnoside inhibits inflammatory responses via MAPK and NF-*κ*B pathways in vitro and in vivo. *Toxicology and Applied Pharmacology*.

[30] Jiang J., Meng Y., Hu S., Botchway B. O. A., Zhang Y., Liu X. (2020). Saikosaponin D: a potential therapeutic drug for osteoarthritis. *Journal of Tissue Engineering and Regenerative Medicine*.

[31] Ahmad Khan M., Sarwar A. H. M. G., Rahat R., Ahmed R. S., Umar S. (2020). Stigmasterol protects rats from collagen induced arthritis by inhibiting proinflammatory cytokines. *International Immunopharmacology*.

[32] Ren X., Han L., Li Y. (2021). Isorhamnetin attenuates TNF-*α*-induced inflammation, proliferation, and migration in human bronchial epithelial cells via MAPK and NF-*κ*B pathways. *The Anatomical Record*.

[33] Fischer R., Kontermann R. E., Pfizenmaier K. (2020). Selective targeting of TNF receptors as a novel therapeutic approach. *Frontiers in Cell and Development Biology*.

[34] Idriss H. T., Naismith J. H. (2000). TNF alpha and the TNF receptor superfamily: structure-function relationship(s). *Microscopy Research and Technique*.

[35] Lee I. T., Lee C. W., Tung W. H. (2010). Cooperation of TLR2 with MyD88, PI3K, and Rac1 in lipoteichoic acid-induced cPLA_2_/COX-2-dependent airway inflammatory responses. *The American Journal of Pathology*.

[36] Yeung Y. T., Aziz F., Guerrero-Castilla A., Arguelles S. (2018). Signaling pathways in inflammation and anti-inflammatory therapies. *Current Pharmaceutical Design*.

[37] Ruiz-Romeu E., Ferran M., Sagristà M. (2016). _Streptococcus pyogenes_ -induced cutaneous lymphocyte antigen-positive T cell-dependent epidermal cell activation triggers T_H_17 responses in patients with guttate psoriasis. *Journal of Allergy and Clinical Immunology*.

[38] Wang B., Dileepan T., Briscoe S. (2010). Induction of TGF-beta1 and TGF-beta1-dependent predominant Th17 differentiation by group A streptococcal infection. *Proceedings of the National Academy of Sciences of the United States of America*.

[39] Miossec P., Kolls J. K. (2012). Targeting IL-17 and T_H_17 cells in chronic inflammation. *Nature Reviews Drug Discovery*.

[40] Beringer A., Noack M., Miossec P. (2016). IL-17 in chronic inflammation: from discovery to targeting. *Trends in Molecular Medicine*.

[41] Yang Y. J., Xi Z. W. (2021). Effects of saikosaponin A on serum interleukin-6 and tumor necrosis factor-*α* after anorectal surgery in rats. *The Chinese Journal of Clinical Pharmacology*.

[42] Lu Z. B., Wang X. Y., Tian S. J., Zhu J. G., Bai Y. D., Gao Y. (2021). Mechanism of quercetin affecting LPS-induced paranasal sinusitis in mice by regulating golgi stress. *Journal of Guizhou Medical University*.

[43] Cavalcanti M. R. M., Passos F. R. S., Monteiro B. S. (2021). HPLC-DAD-UV analysis, anti-inflammatory and anti-neuropathic effects of methanolic extract of Sideritis bilgeriana (lamiaceae) by NF-*κ*B, TNF-*α*, IL-1*β* and IL-6 involvement. *Journal of Ethnopharmacology*.

