# Supplementary material for: Network Pharmacological Analysis and Experimental Study of the Antipharyngitis Mechanism of the Chaiqin Qingning Capsule
Source: Biomed Res Int. 2022 Apr 28;2022:5616942. doi: 10.1155/2022/5616942 (PMC9071881; doi:10.1155/2022/5616942)
Supplement: Supplementary Materials — Supplementary Table 1: information of predicted CQQN targets. Supplementary Table 2: information of known pharyngitis-related targets. Supplementary Table 3: top 10 GO enrichment of biological processes (BP), cellular components (CC), and molecular functions (MF) with their representative enriched terms (one per cluster). Supplementary Table 4: top 20 KEGG pathway. [file 5616942.f1.docx]

**Based on network pharmacology and acute pharyngitis rat model to investigate the effect of Chaiqin Qingning Capsule in treating pharyngitis**

Can Wang^1^, Hongjin Gao^1^, Lianzhan Huang^1^, Zhen Wang^1^, Xuanshneg Ding^1#^.

1: Department of Pharmacy, School of Basic Medicine and Clinical Pharmacy, China Pharmaceutical University, Nanjing 211198, China

**Supplementary Table 1 Information of predicted CQQN targets**

| **Mol ID** | **Molecule Name** | **Target Name** | **Gene names** |
| --- | --- | --- | --- |
| MOL001645 | Linoleyl acetate | Prostaglandin G/H synthase 1 | PTGS1 |
| MOL001645 | Linoleyl acetate | Prostaglandin G/H synthase 2 | PTGS2 |
| MOL001645 | Linoleyl acetate | Nuclear receptor coactivator 2 | NCOA2 |
| MOL001645 | Linoleyl acetate | Retinoic acid receptor RXR-alpha | RXRA |
| MOL002776 | Baicalin | Coagulation factor X | F10 |
| MOL002776 | Baicalin | Tyrosine-protein phosphatase non-receptor type 1 | PTPN6 |
| MOL000449 | Stigmasterol | Progesterone receptor | PGR |
| MOL000449 | Stigmasterol | Mineralocorticoid receptor | NR3C2 |
| MOL000449 | Stigmasterol | Nuclear receptor coactivator 2 | NCOA2 |
| MOL000449 | Stigmasterol | Alcohol dehydrogenase 1C | ADH1C |
| MOL000449 | Stigmasterol | Ig gamma-1 chain C region | IGHG1 |
| MOL000449 | Stigmasterol | Retinoic acid receptor RXR-alpha | RXRA |
| MOL000449 | Stigmasterol | Nuclear receptor coactivator 1 | NCOA1 |
| MOL000449 | Stigmasterol | Prostaglandin G/H synthase 1 | PTGS1 |
| MOL000449 | Stigmasterol | Prostaglandin G/H synthase 2 | PTGS2 |
| MOL000449 | Stigmasterol | Alpha-2A adrenergic receptor | ADRA2A |
| MOL000449 | Stigmasterol | Sodium-dependent noradrenaline transporter | SLC6A2 |
| MOL000449 | Stigmasterol | Sodium-dependent dopamine transporter | SLC6A3 |
| MOL000449 | Stigmasterol | Beta-2 adrenergic receptor | ADRB2 |
| MOL000449 | Stigmasterol | Aldose reductase | AKR1B1 |
| MOL000449 | Stigmasterol | Urokinase-type plasminogen activator | PLAU |
| MOL000449 | Stigmasterol | Leukotriene A-4 hydrolase | LTA4H |
| MOL000449 | Stigmasterol | Amine oxidase [flavin-containing] B | MAOB |
| MOL000449 | Stigmasterol | Amine oxidase [flavin-containing] A | MAOA |
| MOL000449 | Stigmasterol | cAMP-dependent protein kinase catalytic subunit alpha | PRKACA |
| MOL000449 | Stigmasterol | Chymotrypsinogen B | CTRB1 |
| MOL000449 | Stigmasterol | Muscarinic acetylcholine receptor M3 | CHRM3 |
| MOL000449 | Stigmasterol | Muscarinic acetylcholine receptor M1 | CHRM1 |
| MOL000449 | Stigmasterol | Beta-1 adrenergic receptor | ADRB1 |
|  |  |  |  |
| MOL000449 | Stigmasterol | Sodium channel protein type 5 subunit alpha | SCN5A |
| MOL000449 | Stigmasterol | 5-hydroxytryptamine 2A receptor | HTR2A |
| MOL000449 | Stigmasterol | Alpha-1A adrenergic receptor | ADRA1A |
| MOL000449 | Stigmasterol | Gamma-aminobutyric-acid receptor subunit alpha-3 | GABRA3 |
| MOL000449 | Stigmasterol | Muscarinic acetylcholine receptor M2 | CHRM2 |
| MOL000449 | Stigmasterol | Alpha-1B adrenergic receptor | ADRA1B |
| MOL000449 | Stigmasterol | Gamma-aminobutyric-acid receptor subunit alpha-1 | GABRA1 |
| MOL000449 | Stigmasterol | Neuronal acetylcholine receptor subunit alpha-7 | CHRNA7 |
| MOL000354 | isorhamnetin | Nitric oxide synthase, inducible | NOS2 |
| MOL000354 | isorhamnetin | Prostaglandin G/H synthase 1 | PTGS1 |
| MOL000354 | isorhamnetin | Estrogen receptor | ESR1 |
| MOL000354 | isorhamnetin | Androgen receptor | AR |
| MOL000354 | isorhamnetin | Peroxisome proliferator-activated receptor gamma | PPARG |
| MOL000354 | isorhamnetin | Prostaglandin G/H synthase 2 | PTGS2 |
| MOL000354 | isorhamnetin | Tyrosine-protein phosphatase non-receptor type 1 | PTPN6 |
| MOL000354 | isorhamnetin | Estrogen receptor beta | ESR2 |
| MOL000354 | isorhamnetin | Dipeptidyl peptidase 4 | DPP4 |
| MOL000354 | isorhamnetin | Mitogen-activated protein kinase 14 | MAPK14 |
| MOL000354 | isorhamnetin | Glycogen synthase kinase-3 beta | GSK3B |
| MOL000354 | isorhamnetin | Heat shock protein HSP 90-alpha | HSP90AA1 |
| MOL000354 | isorhamnetin | Cell division protein kinase 2 | CDK2 |
| MOL000354 | isorhamnetin | Phosphatidylinositol-4,5-bisphosphate 3-kinase catalytic subunit gamma isoform | PIK3CG |
| MOL000354 | isorhamnetin | cAMP-dependent protein kinase catalytic subunit alpha | PRKACA |
| MOL000354 | isorhamnetin | Trypsin-1 | PRSS1 |
| MOL000354 | isorhamnetin | Proto-oncogene serine/threonine-protein kinase Pim-1 | PIM1 |
| MOL000354 | isorhamnetin | Cyclin-A2 | CCNA2 |
| MOL000354 | isorhamnetin | Nuclear receptor coactivator 2 | NCOA2 |
| MOL000354 | isorhamnetin | Glycogen phosphorylase, muscle form | PYGM |
| MOL000354 | isorhamnetin | Peroxisome proliferator-activated receptor delta | PPARD |
| MOL000354 | isorhamnetin | Serine/threonine-protein kinase Chk1 | CHEK1 |
| MOL000354 | isorhamnetin | Aldose reductase | AKR1B1 |
| MOL000354 | isorhamnetin | Nuclear receptor coactivator 1 | NCOA1 |
| MOL000354 | isorhamnetin | Coagulation factor VII | F7 |
| MOL000354 | isorhamnetin | Prothrombin | F2 |
| MOL000354 | isorhamnetin | Nitric-oxide synthase, endothelial | NOS3 |
| MOL000354 | isorhamnetin | Acetylcholinesterase | ACHE |
| MOL000354 | isorhamnetin | Gamma-aminobutyric-acid receptor subunit alpha-1 | GABRA1 |
| MOL000354 | isorhamnetin | Amine oxidase [flavin-containing] B | MAOB |
| MOL000354 | isorhamnetin | Glutamate receptor 2 | GRIA2 |
| MOL000354 | isorhamnetin | Transcription factor p65 | RELA |
| MOL000354 | isorhamnetin | Xanthine dehydrogenase/oxidase | XDH |
| MOL000354 | isorhamnetin | Neutrophil cytosol factor 1 | NCF1 |
| MOL000354 | isorhamnetin | Oxidized low-density lipoprotein receptor 1 | OLR1 |
| MOL000422 | kaempferol | Nitric oxide synthase, inducible | NOS2 |
| MOL000422 | kaempferol | Prostaglandin G/H synthase 1 | PTGS1 |
| MOL000422 | kaempferol | Androgen receptor | AR |
| MOL000422 | kaempferol | Peroxisome proliferator-activated receptor gamma | PPARG |
| MOL000422 | kaempferol | Prostaglandin G/H synthase 2 | PTGS2 |
| MOL000422 | kaempferol | Heat shock protein HSP 90-alpha | HSP90AA1 |
| MOL000422 | kaempferol | Phosphatidylinositol-4,5-bisphosphate 3-kinase catalytic subunit gamma isoform | PIK3CG |
| MOL000422 | kaempferol | cAMP-dependent protein kinase catalytic subunit alpha | PRKACA |
| MOL000422 | kaempferol | Nuclear receptor coactivator 2 | NCOA2 |
| MOL000422 | kaempferol | Dipeptidyl peptidase 4 | DPP4 |
| MOL000422 | kaempferol | Trypsin-1 | PRSS1 |
| MOL000422 | kaempferol | Progesterone receptor | PGR |
| MOL000422 | kaempferol | Prothrombin | F2 |
| MOL000422 | kaempferol | Muscarinic acetylcholine receptor M1 | CHRM1 |
| MOL000422 | kaempferol | Nitric-oxide synthase, endothelial | NOS3 |
| MOL000422 | kaempferol | Gamma-aminobutyric-acid receptor subunit alpha-2 | GABRA2 |
| MOL000422 | kaempferol | Acetylcholinesterase | ACHE |
| MOL000422 | kaempferol | Sodium-dependent noradrenaline transporter | SLC6A2 |
| MOL000422 | kaempferol | Muscarinic acetylcholine receptor M2 | CHRM2 |
| MOL000422 | kaempferol | Alpha-1B adrenergic receptor | ADRA1B |
| MOL000422 | kaempferol | Gamma-aminobutyric-acid receptor subunit alpha-1 | GABRA1 |
| MOL000422 | kaempferol | DNA topoisomerase 2-alpha | TOP2A |
| MOL000422 | kaempferol | Coagulation factor VII | F7 |
| MOL000422 | kaempferol | Transcription factor p65 | RELA |
| MOL000422 | kaempferol | Inhibitor of nuclear factor kappa-B kinase subunit beta | IKBKB |
| MOL000422 | kaempferol | RAC-alpha serine/threonine-protein kinase | AKT1 |
| MOL000422 | kaempferol | Apoptosis regulator Bcl-2 | BCL2 |
| MOL000422 | kaempferol | Apoptosis regulator BAX | BAX |
| MOL000422 | kaempferol | Tumor necrosis factor | TNF |
| MOL000422 | kaempferol | Transcription factor AP-1 | JUN |
| MOL000422 | kaempferol | Activator of 90 kDa heat shock protein ATPase homolog 1 | AHSA1 |
| MOL000422 | kaempferol | Caspase-3 | CASP3 |
| MOL000422 | kaempferol | Mitogen-activated protein kinase 8 | MAPK8 |
| MOL000422 | kaempferol | Xanthine dehydrogenase/oxidase | XDH |
| MOL000422 | kaempferol | Interstitial collagenase | MMP1 |
| MOL000422 | kaempferol | Signal transducer and activator of transcription 1-alpha/beta | STAT1 |
| MOL000422 | kaempferol | Cell division control protein 2 homolog | CDK1 |
| MOL000422 | kaempferol | Peroxisome proliferator-activated receptor gamma | PPARG |
| MOL000422 | kaempferol | Heme oxygenase 1 | HMOX1 |
| MOL000422 | kaempferol | Cytochrome P450 3A4 | CYP3A4 |
| MOL000422 | kaempferol | Cytochrome P450 1A2 | CYP1A2 |
| MOL000422 | kaempferol | Cytochrome P450 1A1 | CYP1A1 |
| MOL000422 | kaempferol | Intercellular adhesion molecule 1 | ICAM1 |
| MOL000422 | kaempferol | E-selectin | SELE |
| MOL000422 | kaempferol | Vascular cell adhesion protein 1 | VCAM1 |
| MOL000422 | kaempferol | Nuclear receptor subfamily 1 group I member 2 | NR1I2 |
| MOL000422 | kaempferol | Cytochrome P450 1B1 | CYP1B1 |
| MOL000422 | kaempferol | Arachidonate 5-lipoxygenase | ALOX5 |
| MOL000422 | kaempferol | Hyaluronan synthase 2 | HAS2 |
| MOL000422 | kaempferol | Aryl hydrocarbon receptor | AHR |
| MOL000422 | kaempferol | 26S proteasome non-ATPase regulatory subunit 3 | PSMD3 |
| MOL000422 | kaempferol | Solute carrier family 2, facilitated glucose transporter member 4 | SLC2A4 |
| MOL000422 | kaempferol | Nuclear receptor subfamily 1 group I member 3 | NR1I3 |
| MOL000422 | kaempferol | Insulin receptor | INSR |
| MOL000422 | kaempferol | Type I iodothyronine deiodinase | DIO1 |
| MOL000422 | kaempferol | Serine/threonine-protein phosphatase 2B catalytic subunit alpha isoform | PPP3CA |
| MOL000422 | kaempferol | Glutathione S-transferase Mu 1 | GSTM1 |
| MOL000422 | kaempferol | Glutathione S-transferase Mu 2 | GSTM2 |
| MOL000422 | kaempferol | Aldo-keto reductase family 1 member C3 | AKR1C3 |
| MOL004598 | kaempferol | Antileukoproteinase | SLPI |
| MOL004598 | 3,5,6,7-tetramethoxy-2-(3,4,5-trimethoxyphenyl)chromone | Prothrombin | F2 |
| MOL004598 | 3,5,6,7-tetramethoxy-2-(3,4,5-trimethoxyphenyl)chromone | Estrogen receptor | ESR1 |
| MOL004598 | 3,5,6,7-tetramethoxy-2-(3,4,5-trimethoxyphenyl)chromone | Androgen receptor | AR |
| MOL004598 | 3,5,6,7-tetramethoxy-2-(3,4,5-trimethoxyphenyl)chromone | Coagulation factor X | F10 |
| MOL004598 | 3,5,6,7-tetramethoxy-2-(3,4,5-trimethoxyphenyl)chromone | Prostaglandin G/H synthase 2 | PTGS2 |
| MOL004598 | 3,5,6,7-tetramethoxy-2-(3,4,5-trimethoxyphenyl)chromone | Coagulation factor VII | F7 |
| MOL004598 | 3,5,6,7-tetramethoxy-2-(3,4,5-trimethoxyphenyl)chromone | Acetylcholinesterase | ACHE |
| MOL004598 | 3,5,6,7-tetramethoxy-2-(3,4,5-trimethoxyphenyl)chromone | DNA topoisomerase 2-alpha | TOP2A |
| MOL004598 | 3,5,6,7-tetramethoxy-2-(3,4,5-trimethoxyphenyl)chromone | Estrogen receptor beta | ESR2 |
| MOL004598 | 3,5,6,7-tetramethoxy-2-(3,4,5-trimethoxyphenyl)chromone | Trypsin-1 | PRSS1 |
| MOL004598 | 3,5,6,7-tetramethoxy-2-(3,4,5-trimethoxyphenyl)chromone | Nuclear receptor coactivator 2 | NCOA2 |
| MOL004609 | Areapillin | Nitric oxide synthase, inducible | NOS2 |
| MOL004609 | Areapillin | Prothrombin | F2 |
| MOL004609 | Areapillin | Androgen receptor | AR |
| MOL004609 | Areapillin | Sodium channel protein type 5 subunit alpha | SCN5A |
| MOL004609 | Areapillin | Coagulation factor X | F10 |
| MOL004609 | Areapillin | Prostaglandin G/H synthase 2 | PTGS2 |
| MOL004609 | Areapillin | Coagulation factor VII | F7 |
| MOL004609 | Areapillin | Tyrosine-protein phosphatase non-receptor type 1 | PTPN6 |
| MOL004609 | Areapillin | DNA topoisomerase 2-alpha | TOP2A |
| MOL004609 | Areapillin | Estrogen receptor beta | ESR2 |
| MOL004609 | Areapillin | Dipeptidyl peptidase 4 | DPP4 |
| MOL004609 | Areapillin | Heat shock protein HSP 90-alpha | HSP90AA1 |
| MOL004609 | Areapillin | Ig gamma-1 chain C region | IGHG1 |
| MOL004609 | Areapillin | Trypsin-1 | PRSS1 |
| MOL004609 | Areapillin | Nuclear receptor coactivator 2 | NCOA2 |
| MOL004609 | Areapillin | Nuclear receptor coactivator 1 | NCOA1 |
| MOL013187 | Cubebin | Prostaglandin G/H synthase 1 | PTGS1 |
| MOL013187 | Cubebin | Coagulation factor X | F10 |
| MOL013187 | Cubebin | Prostaglandin G/H synthase 2 | PTGS2 |
| MOL013187 | Cubebin | Beta-2 adrenergic receptor | ADRB2 |
| MOL004624 | Cubebin | Heat shock protein HSP 90-alpha | HSP90AA1 |
| MOL004624 | Longikaurin A | Muscarinic acetylcholine receptor M1 | CHRM1 |
| MOL004624 | Longikaurin A | Gamma-aminobutyric-acid receptor subunit alpha-2 | GABRA2 |
| MOL004624 | Longikaurin A | Gamma-aminobutyric-acid receptor subunit alpha-3 | GABRA3 |
| MOL004624 | Longikaurin A | Muscarinic acetylcholine receptor M2 | CHRM2 |
| MOL004624 | Longikaurin A | Trypsin-1 | PRSS1 |
| MOL004653 | Longikaurin A | Gamma-aminobutyric-acid receptor subunit alpha-6 | GABRA6 |
| MOL004653 | (+)-Anomalin | Prothrombin | F2 |
| MOL004653 | (+)-Anomalin | Potassium voltage-gated channel subfamily H member 2 | KCNH2 |
| MOL004653 | (+)-Anomalin | Coagulation factor X | F10 |
| MOL004653 | (+)-Anomalin | Prostaglandin G/H synthase 2 | PTGS2 |
| MOL004653 | (+)-Anomalin | DNA topoisomerase 2-alpha | TOP2A |
| MOL004718 | (+)-Anomalin | Dipeptidyl peptidase 4 | DPP4 |
| MOL004718 | α-spinasterol | Progesterone receptor | PGR |
| MOL004718 | α-spinasterol | Mineralocorticoid receptor | NR3C2 |
| MOL000490 | α-spinasterol | Nuclear receptor coactivator 2 | NCOA2 |
| MOL000490 | petunidin | Nitric oxide synthase, inducible | NOS2 |
| MOL000490 | petunidin | Prostaglandin G/H synthase 1 | PTGS1 |
| MOL000490 | petunidin | Prostaglandin G/H synthase 2 | PTGS2 |
| MOL000490 | petunidin | Estrogen receptor beta | ESR2 |
| MOL000490 | petunidin | Mitogen-activated protein kinase 14 | MAPK14 |
| MOL000490 | petunidin | Glycogen synthase kinase-3 beta | GSK3B |
| MOL000490 | petunidin | Heat shock protein HSP 90-alpha | HSP90AA1 |
| MOL000098 | petunidin | Nuclear receptor coactivator 2 | NCOA2 |
| MOL000098 | quercetin | Prostaglandin G/H synthase 1 | PTGS1 |
| MOL000098 | quercetin | Androgen receptor | AR |
| MOL000098 | quercetin | Peroxisome proliferator-activated receptor gamma | PPARG |
| MOL000098 | quercetin | Prostaglandin G/H synthase 2 | PTGS2 |
| MOL000098 | quercetin | Heat shock protein HSP 90-alpha | HSP90AA1 |
| MOL000098 | quercetin | Phosphatidylinositol-4,5-bisphosphate 3-kinase catalytic subunit gamma isoform | PIK3CG |
| MOL000098 | quercetin | Nuclear receptor coactivator 2 | NCOA2 |
| MOL000098 | quercetin | Dipeptidyl peptidase 4 | DPP4 |
| MOL000098 | quercetin | Aldose reductase | AKR1B1 |
| MOL000098 | quercetin | Trypsin-1 | PRSS1 |
| MOL000098 | quercetin | DNA topoisomerase 2-alpha | TOP2A |
| MOL000098 | quercetin | Prothrombin | F2 |
| MOL000098 | quercetin | Potassium voltage-gated channel subfamily H member 2 | KCNH2 |
| MOL000098 | quercetin | Sodium channel protein type 5 subunit alpha | SCN5A |
| MOL000098 | quercetin | Coagulation factor X | F10 |
| MOL000098 | quercetin | Beta-2 adrenergic receptor | ADRB2 |
| MOL000098 | quercetin | Stromelysin-1 | MMP3 |
| MOL000098 | quercetin | cAMP-dependent protein kinase catalytic subunit alpha | PRKACA |
| MOL000098 | quercetin | Coagulation factor VII | F7 |
| MOL000098 | quercetin | Nitric-oxide synthase, endothelial | NOS3 |
| MOL000098 | quercetin | Retinoic acid receptor RXR-alpha | RXRA |
| MOL000098 | quercetin | Acetylcholinesterase | ACHE |
| MOL000098 | quercetin | Gamma-aminobutyric-acid receptor subunit alpha-1 | GABRA1 |
| MOL000098 | quercetin | Amine oxidase [flavin-containing] B | MAOB |
| MOL000098 | quercetin | Transcription factor p65 | RELA |
| MOL000098 | quercetin | Epidermal growth factor receptor | EGFR |
| MOL000098 | quercetin | RAC-alpha serine/threonine-protein kinase | AKT1 |
| MOL000098 | quercetin | Vascular endothelial growth factor A | VEGFA |
| MOL000098 | quercetin | G1/S-specific cyclin-D1 | CCND1 |
| MOL000098 | quercetin | Apoptosis regulator Bcl-2 | BCL2 |
| MOL000098 | quercetin | Bcl-2-like protein 1 | BCL2L1 |
| MOL000098 | quercetin | Proto-oncogene c-Fos | FOS |
| MOL000098 | quercetin | Cyclin-dependent kinase inhibitor 1 | CDKN1A |
| MOL000098 | quercetin | Eukaryotic translation initiation factor 6 | EIF6 |
| MOL000098 | quercetin | Apoptosis regulator BAX | BAX |
| MOL000098 | quercetin | Caspase-9 | CASP9 |
| MOL000098 | quercetin | Urokinase-type plasminogen activator | PLAU |
| MOL000098 | quercetin | 72 kDa type IV collagenase | MMP2 |
| MOL000098 | quercetin | Matrix metalloproteinase-9 | MMP9 |
| MOL000098 | quercetin | Mitogen-activated protein kinase 1 | MAPK1 |
| MOL000098 | quercetin | Interleukin-10 | IL10 |
| MOL000098 | quercetin | Retinoblastoma-associated protein | RB1 |
| MOL000098 | quercetin | Tumor necrosis factor | TNF |
| MOL000098 | quercetin | Transcription factor AP-1 | JUN |
| MOL000098 | quercetin | Interleukin-6 | IL6 |
| MOL000098 | quercetin | Caspase-3 | CASP3 |
| MOL000098 | quercetin | Cellular tumor antigen p53 | TP53 |
| MOL000098 | quercetin | ETS domain-containing protein Elk-1 | ELK1 |
| MOL000098 | quercetin | NF-kappa-B inhibitor alpha | NFKBIA |
| MOL000098 | quercetin | Ornithine decarboxylase | ODC1 |
| MOL000098 | quercetin | Xanthine dehydrogenase/oxidase | XDH |
| MOL000098 | quercetin | Caspase-8 | CASP8 |
| MOL000098 | quercetin | DNA topoisomerase 1 | TOP1 |
| MOL000098 | quercetin | RAF proto-oncogene serine/threonine-protein kinase | RAF1 |
| MOL000098 | quercetin | Superoxide dismutase [Cu-Zn] | SOD1 |
| MOL000098 | quercetin | Protein kinase C alpha type | PRKCA |
| MOL000098 | quercetin | Interstitial collagenase | MMP1 |
| MOL000098 | quercetin | Hypoxia-inducible factor 1-alpha | HIF1A |
| MOL000098 | quercetin | Signal transducer and activator of transcription 1-alpha/beta | STAT1 |
| MOL000098 | quercetin | Protein CBFA2T1 | RUNX1T1 |
| MOL000098 | quercetin | 78 kDa glucose-regulated protein | HSPA5 |
| MOL000098 | quercetin | Receptor tyrosine-protein kinase erbB-2 | ERBB2 |
| MOL000098 | quercetin | Peroxisome proliferator-activated receptor gamma | PPARG |
| MOL000098 | quercetin | Acetyl-CoA carboxylase 1 | ACACA |
| MOL000098 | quercetin | Heme oxygenase 1 | HMOX1 |
| MOL000098 | quercetin | Cytochrome P450 3A4 | CYP3A4 |
| MOL000098 | quercetin | Caveolin-1 | CAV1 |
| MOL000098 | quercetin | Myc proto-oncogene protein | MYC |
| MOL000098 | quercetin | Tissue factor | F3 |
| MOL000098 | quercetin | Gap junction alpha-1 protein | GJA1 |
| MOL000098 | quercetin | Cytochrome P450 1A1 | CYP1A1 |
| MOL000098 | quercetin | Intercellular adhesion molecule 1 | ICAM1 |
| MOL000098 | quercetin | Interleukin-1 beta | IL1B |
| MOL000098 | quercetin | E-selectin | SELE |
| MOL000098 | quercetin | Vascular cell adhesion protein 1 | VCAM1 |
| MOL000098 | quercetin | Prostaglandin E2 receptor, EP3 subtype | PTGER3 |
| MOL000098 | quercetin | Interleukin-8 | CXCL8 |
| MOL000098 | quercetin | Protein kinase C beta type | PRKCB |
| MOL000098 | quercetin | Baculoviral IAP repeat-containing protein 5 | BIRC5 |
| MOL000098 | quercetin | Dual oxidase 2 | DUOX2 |
| MOL000098 | quercetin | Nitric oxide synthase, endothelial | NOS3 |
| MOL000098 | quercetin | Heat shock protein beta-1 | HSPB1 |
| MOL000098 | quercetin | Interleukin-2 | IL2 |
| MOL000098 | quercetin | Nuclear receptor subfamily 1 group I member 2 | NR1I2 |
| MOL000098 | quercetin | Cytochrome P450 1B1 | CYP1B1 |
| MOL000098 | quercetin | G2/mitotic-specific cyclin-B1 | CCNB1 |
| MOL000098 | quercetin | Tissue-type plasminogen activator | PLAT |
| MOL000098 | quercetin | Thrombomodulin | THBD |
| MOL000098 | quercetin | Plasminogen activator inhibitor 1 | SERPINE1 |
| MOL000098 | quercetin | Interferon gamma | IFNG |
| MOL000098 | quercetin | Arachidonate 5-lipoxygenase | ALOX5 |
| MOL000098 | quercetin | Interleukin-1 alpha | IL1A |
| MOL000098 | quercetin | Myeloperoxidase | MPO |
| MOL000098 | quercetin | DNA topoisomerase 2-alpha | TOP2A |
| MOL000098 | quercetin | Neutrophil cytosol factor 1 | NCF1 |
| MOL000098 | quercetin | ATP-binding cassette sub-family G member 2 | ABCG2 |
| MOL000098 | quercetin | Hyaluronan synthase 2 | HAS2 |
| MOL000098 | quercetin | Nuclear factor erythroid 2-related factor 2 | NFE2L2 |
| MOL000098 | quercetin | NAD(P)H dehydrogenase [quinone] 1 | NQO1 |
| MOL000098 | quercetin | Poly [ADP-ribose] polymerase 1 | PARP1 |
| MOL000098 | quercetin | Aryl hydrocarbon receptor | AHR |
| MOL000098 | quercetin | 26S proteasome non-ATPase regulatory subunit 3 | PSMD3 |
| MOL000098 | quercetin | Solute carrier family 2, facilitated glucose transporter member 4 | SLC2A4 |
| MOL000098 | quercetin | Collagen alpha-1(III) chain | COL3A1 |
| MOL000098 | quercetin | C-X-C motif chemokine 11 | CXCL11 |
| MOL000098 | quercetin | C-X-C motif chemokine 2 | CXCL2 |
| MOL000098 | quercetin | DDB1- and CUL4-associated factor 5 | DCAF5 |
| MOL000098 | quercetin | Nuclear receptor subfamily 1 group I member 3 | NR1I3 |
| MOL000098 | quercetin | Serine/threonine-protein kinase Chk2 | CHEK2 |
| MOL000098 | quercetin | Insulin receptor | INSR |
| MOL000098 | quercetin | Claudin-4 | CLDN4 |
| MOL000098 | quercetin | Peroxisome proliferator-activated receptor alpha | PPARA |
| MOL000098 | quercetin | Peroxisome proliferator-activated receptor delta | PPARD |
| MOL000098 | quercetin | Heat shock factor protein 1 | HSF1 |
| MOL000098 | quercetin | C-X-C motif chemokine 10 | CXCL10 |
| MOL000098 | quercetin | Inhibitor of nuclear factor kappa-B kinase subunit alpha | CHUK |
| MOL000098 | quercetin | Osteopontin | SPP1 |
| MOL000098 | quercetin | Runt-related transcription factor 2 | RUNX2 |
| MOL000098 | quercetin | Ras association domain-containing protein 1 | RASSF1 |
| MOL000098 | quercetin | Transcription factor E2F1 | E2F1 |
| MOL000098 | quercetin | Transcription factor E2F2 | E2F2 |
| MOL000098 | quercetin | Prostatic acid phosphatase | ACP3 |
| MOL000098 | quercetin | Cathepsin D | CTSD |
| MOL000098 | quercetin | Insulin-like growth factor-binding protein 3 | IGFBP3 |
| MOL000098 | quercetin | Insulin-like growth factor II | IGF2 |
| MOL000098 | quercetin | CD40 ligand | CD40LG |
| MOL000098 | quercetin | Interferon regulatory factor 1 | IRF1 |
| MOL000098 | quercetin | Receptor tyrosine-protein kinase erbB-3 | ERBB3 |
| MOL000098 | quercetin | Serum paraoxonase/arylesterase 1 | PON1 |
| MOL000098 | quercetin | Type I iodothyronine deiodinase | DIO1 |
| MOL000098 | quercetin | Procollagen C-endopeptidase enhancer 1 | PCOLCE |
| MOL000098 | quercetin | Puromycin-sensitive aminopeptidase | NPEPPS |
| MOL000098 | quercetin | Hexokinase-2 | HK2 |
| MOL000098 | quercetin | Homeobox protein Nkx-3.1 | NKX3-1 |
| MOL000098 | quercetin | Ras GTPase-activating protein 1 | RASA1 |
| MOL000098 | quercetin | Glutathione S-transferase Mu 1 | GSTM1 |
| MOL000098 | quercetin | Glutathione S-transferase Mu 2 | GSTM2 |
| MOL004635 | Saikosaponin A | Interleukin-2 | IL2 |
| MOL004636 | Saikosaponin A | Signal transducer and activator of transcription 3 | STAT3 |
| MOL004637 | Saikosaponin A | Platelet-activating factor receptor | PTAFR |
| MOL004638 | Saikosaponin A | Serine/threonine-protein kinase mTOR | MTOR |
| MOL004639 | Saikosaponin A | Tyrosine-protein phosphatase non-receptor type 1 | PTPN1 |
| MOL004640 | Saikosaponin A | Adenosine receptor A1 | ADORA1 |
| MOL004641 | Saikosaponin A | Tyrosine-protein kinase SYK | SYK |
| MOL004642 | Saikosaponin A | Nuclear receptor ROR-gamma | RORC |
| MOL004643 | Saikosaponin A | Leukotriene B4 receptor 1 | LTB4R |
| MOL004644 | Saikosaponin A | Sodium/glucose cotransporter 2 | SLC5A2 |
| MOL004645 | Saikosaponin A | Sodium/glucose cotransporter 1 | SLC5A1 |
| MOL004646 | Saikosaponin A | Delta-type opioid receptor | OPRD1 |
| MOL004647 | Saikosaponin A | Metabotropic glutamate receptor 2 | GRM2 |
| MOL004648 | Saikosaponin A | cGMP-specific 3',5'-cyclic phosphodiesterase | PDE5A |
| MOL004649 | Saikosaponin A | Serine/threonine-protein phosphatase PP1-gamma catalytic subunit | PPP1CC |
| MOL004650 | Saikosaponin A | Tyrosine-protein phosphatase non-receptor type 2 | PTPN2 |
| MOL004651 | Saikosaponin A | Muscarinic acetylcholine receptor M4 | CHRM4 |
| MOL004652 | Saikosaponin A | Muscarinic acetylcholine receptor M5 | CHRM5 |
| MOL004653 | Saikosaponin A | Muscarinic acetylcholine receptor M2 | CHRM2 |
| MOL004654 | Saikosaponin A | Muscarinic acetylcholine receptor M1 | CHRM1 |
| MOL004655 | Saikosaponin A | Muscarinic acetylcholine receptor M3 | CHRM3 |
| MOL004656 | Saikosaponin A | Adenosine receptor A2b | ADORA2B |
| MOL004657 | Saikosaponin A | Adenosine receptor A3 | ADORA3 |
| MOL004658 | Saikosaponin a | Bcl-2-like protein 1 | BCL2L1 |
| MOL004636 | Saikosaponin B | Signal transducer and activator of transcription 3 | STAT3 |
| MOL004637 | Saikosaponin B | Interleukin-2 | IL2 |
| MOL004638 | Saikosaponin B | Platelet-activating factor receptor | PTAFR |
| MOL004639 | Saikosaponin B | Tyrosine-protein phosphatase non-receptor type 22 | PTPN22 |
| MOL004640 | Saikosaponin B | Thymidylate synthase | TYMS |
| MOL004701 | Saikosaponin C | Platelet-activating factor receptor | PTAFR |
| MOL004702 | Saikosaponin C | Nuclear receptor ROR-gamma | RORC |
| MOL004703 | Saikosaponin C | Signal transducer and activator of transcription 3 | STAT3 |
| MOL004704 | Saikosaponin C | Tyrosine-protein phosphatase non-receptor type 1 | PTPN1 |
| MOL004705 | Saikosaponin C | Sodium/potassium-transporting ATPase subunit alpha-1 | ATP1A1 |
| MOL004706 | Saikosaponin C | Protein phosphatase 1B | PPM1B |
| MOL004707 | Saikosaponin C | Serine/threonine-protein phosphatase PP1-gamma catalytic subunit | PPP1CC |
| MOL004708 | Saikosaponin C | Serine/threonine-protein phosphatase 2A catalytic subunit alpha isoform | PPP2CA |
| MOL004709 | Saikosaponin C | Serine/threonine-protein phosphatase 2A 56 kDa regulatory subunit alpha isoform | PPP2R5A |
| MOL004710 | Saikosaponin C | Corticosteroid 11-beta-dehydrogenase isozyme 2 | HSD11B2 |
| MOL004637 | Saikosaponin D | Signal transducer and activator of transcription 3 | STAT3 |
| MOL004638 | Saikosaponin D | Interleukin-2 | IL2 |
| MOL004639 | Saikosaponin D | Tyrosine-protein phosphatase non-receptor type 1 | PTPN1 |
| MOL004640 | Saikosaponin D | Platelet-activating factor receptor | PTAFR |
| MOL004641 | Saikosaponin D | Renin (EC 3.4.23.15) | REN |
| MOL004642 | Saikosaponin D | Hexokinase-4 (HK4) (EC 2.7.1.1) | GCK |
| MOL004643 | Saikosaponin D | Leukotriene B4 receptor 1 | LTB4R |
| MOL004644 | Saikosaponin D | Tyrosine-protein phosphatase non-receptor type 2 | PTPN2 |
| MOL004645 | Saikosaponin D | Receptor-type tyrosine-protein phosphatase alpha | PTPRA |
| MOL004646 | Saikosaponin D | Adenosine receptor A1 | ADORA1 |
| MOL004647 | Saikosaponin D | Epidermal growth factor receptor | EGFR |
| MOL004648 | Saikosaponin D | Beta-secretase 1 | BACE1 |
| MOL004649 | Saikosaponin D | Thymidylate synthase | TYMS |
| MOL004650 | Saikosaponin D | Tyrosine-protein phosphatase non-receptor type 22 | PTPN22 |
| MOL004651 | Saikosaponin D | Sodium/glucose cotransporter 2 | SLC5A2 |
| MOL004652 | Saikosaponin D | Sodium/glucose cotransporter 1 | SLC5A1 |

**Supplementary Table 2** **Information of known pharyngitis-related targets**

| **Target names** | **Target names** | **Target names** |
| --- | --- | --- |
| TCIRG1 | STXBP1 | MIR22 |
| SRP54 | CTNS | MIR1306 |
| GFI1 | TAOK1 | DGS2 |
| CXCR4 | TLR3 | CTTN |
| GALNS | GSTP1 | MIR145 |
| GAST | EGFR | IL2RG |
| ELANE | MIR132 | DCLRE1C |
| PAGR1 | PPP3CA | IGHM |
| MYOM2 | CSNK2B | CP |
| FN1 | CAMK2A | CSF3 |
| WDHD1 | CAMK2B | CASP3 |
| RHD | SLC2A3 | SELE |
| SAG | ADCY6 | AASS |
| TNF | YY1 | EBAG9 |
| UGCG | RAB11A | PTH |
| NLRP12 | EEF1A2 | MIR330 |
| GGCT | CLTC | RASSF1 |
| TLR9 | TCF4 | SYP |
| PDR | SET | PPL |
| SLC9A6 | SERPING1 | IFIH1 |
| BATF2 | ASPA | IFNB1 |
| CYP2C19 | DYNC1H1 | CDK4 |
| SUMF2 | CDH15 | PGR |
| AMCN | CACNG2 | MIR199A1 |
| GCLC | SYNGAP1 | MTHFR |
| HLA-C | NGLY1 | SNAI1 |
| IL6 | KDM5B | POF1B |
| MEFV | CUX1 | DSP |
| OTC | UBB | TNC |
| CD6 | SEPSECS | IL18 |
| SCN2A | KCNQ5 | NRP1 |
| SCN1A | KIRREL3 | CHAF1A |
| ABCB1 | KIF1A | EPHX1 |
| CYP3A4 | BRSK2 | ATXN2 |
| PADI4 | CNTNAP1 | MUC1 |
| ABCC2 | CIC | CD274 |
| SCN10A | EPB41L1 | UBE3A |
| ABCB1 | TRPM3 | NAT2 |
| CES1 | FLCN | MIR200A |
| CYP1A2 | ZMYND11 | HAND2 |
| CYP2B6 | ASH1L | LOC102724770 |
| CYP2D6 | TSEN2 | CD36 |
| CYP2E1 | MYT1L | VCAN |
| ALB | EXOSC9 | CXCL12 |
| ORM1 | DEAF1 | ACE |
| pbpA | TSEN15 | STAT2 |
| ftsI | RAI1 | ICOSLG |
| SLC15A1 | GRHL3 | NFIB |
| SLC15A2 | TSEN34 | MIR143 |
| mrcA | CHAMP1 | MIR141 |
| mrcB | HIVEP2 | MIR30E |
| mrdA | TSEN54 | MIR99A |
| SLC22A6 | MBD5 | MIR149 |
| SLC22A8 | MED12L | MIR193B |
| SLC5A1 | MTRFR | MIR30B |
| acrB | HP | MIR373 |
| XIAP | VEGFA | MIR139 |
| CNGA1 | PRKCD | MIR486-1 |
| KCNMA1 | CD81 | FAM3D-AS1 |
| qacR | MS4A1 | HSPG2 |
| ALOX5 | TNFRSF13B | CREBBP |
| PTGS2 | TNFRSF13C | EP300 |
| LTB4R | ICOS | ENO2 |
| TBXA2R | TNFSF12 | KAT6A |
| UGT1A9 | IL2 | HOXA1 |
| MAOA | IL10 | MMP9 |
| DRD1 | HTR2A | COL11A2 |
| ADRB1 | GABRA2 | HOXB1 |
| ADRB2 | CYP1A1 | TGFB1 |
| ADRA2A | NKX2-5 | COL2A1 |
| ACHE | PLG | VAPB |
| GLB1 | RAG2 | FUS |
| ADRA2B | HLA-DQA1 | PRPH |
| ADRA2C | IKZF1 | FIG4 |
| ORM2 | SLC34A1 | SETX |
| CPT2 | EDAR | CAMK2N2 |
| IL6R | FAS | MIR192 |
| FCGR2A | GSTT1 | SNHG4 |
| RANBP2 | MIR9-1 | CASP8 |
| IL1RN | CFHR1 | HSPA4 |
| STAT1 | MGMT | FZD4 |
| MTBS1 | ACP1 | CASR |
| NRAMP1 | SNCA | LRP5 |
| CX3CR1 | MYOT | DICER1 |
| CCR2 | RIPPLY3 | DVL1 |
| CCR5 | CCNA2 | TBX3 |
| IL6ST | SPECC1L | DROSHA |
| PFBI | CCR6 | XPO5 |
| SM1 | TLR2 | AGO2 |
| SM2 | GIPC1 | GCM2 |
| IFNGR1 | ALDH1A2 | SMTN |
| NUP214 | MIR185 | AIFM3 |
| MBL2 | BDNF | BFAR |
| UNC93B1 | TTR | RNH1 |
| TBK1 | CD44 | GTF3A |
| SNORA31 | RET | TBX10 |
| IRF9 | TNFRSF10B | TSSK2 |
| TRAF3 | NF1 | YPEL1 |
| CCL3 | MDH2 | YPEL2 |
| STAT3 | MAX | RMRP |
| TICAM1 | SDHA | MIR200B |
| IFNL3 | DLST | MIR24-1 |
| FUT2 | VHL | MIR15B |
| IRF3 | FH | GGT3P |
| ZNF341 | KIF1B | MIR183 |
| STK4 | SDHD | DGCR11 |
| RPGR | SDHC | MIR502 |
| MAGT1 | ING1 | MIR20B |
| MATR3 | SLC25A11 | FAM230A |
| MIR452 | SDHAF2 | DVL1P1 |
| TBX1 | ING3 | DGCR12 |
| COMT | CFHR5 | FRAXA |
| LACTB | TMEM127 | ENSG00000273032 |
| HIRA | MIR210 | ENSG00000281530 |
| UFD1 | MIR205 | ENSG00000224836 |
| GP1BB | MIR98 | LOC108449897 |
| ARVCF | SPECC1L-ADORA2A | LOC116309126 |
| DGCR6 | LOC100506321 | LOC108449888 |
| LRP12 | LOC107303340 | BLK |
| ADH1C | DRD2 | FYN |
| IL1B | FTL | USP22 |
| COL11A1 | WNT10A | MPO |
| SEC24C | ARID1B | SLC1A2 |
| RREB1 | EDA2R | TLR8 |
| JMJD1C | LEP | NPY |
| TP53 | CCND1 | F3 |
| TANGO2 | CXCL9 | IL1A |
| CRP | TLR4 | MIR140 |
| BMP4 | CALCA | MIR490 |
| HLA-DRB1 | CD40LG | ERBB2 |
| PABPN1 | PIK3CA | DMAP1 |
| CXCL8 | RAG1 | AKR1A1 |
| ADH5 | IL7 | NOD2 |
| CD4 | CTLA4 | MMP11 |
| CSF2 | CHD7 | CCL2 |
| CD8A | MED15 | ADH1A |
| FCGR1A | FMR1 | PDPN |
| NKX2-6 | MRPL40 | MYC |
| DGCR8 | MAOB | CTNNB1 |
| DGCR2 | HIF1A | TGFBR2 |
| ESS2 | ATM | CSF3R |
| SH2D1A | GSC | PDE6A |
| CD1A | DES | COL9A3 |
| NFKB2 | MIR142 | COL9A1 |
| FGF8 | MIR31 | COL9A2 |
| TNFRSF1A | MIR196A1 | FMOD |
| DOCK8 | GATA4 | COL15A1 |
| CARD8 | NRXN1 | ST18 |
| SPAG7 | PRODH | PLEKHA7 |
| ADH1B | GNB1L | SMAP2 |
| GSTM1 | DGCR5 | PCMTD1 |
| CDH1 | FADD | EPDR1 |
| MID1 | CRKL | VIT |
| EDN1 | ATP2B3 | FAM102A |
| NLRP3 | CD27 | TGFBR1 |
| GPT | DDX58 | CYP26A1 |
| MVK | IFNA1 | ACVR1 |
| GRIN1 | SERPINA3 | CSF1 |
| ALS2 | FOXN1 | TLR7 |
| ISL1 | TUBA1A | LCK |
| SDHB | TLR5 | JUP |
| PTPN22 | REV3L | ZEB2 |
| TP63 | KRT13 | LAMC3 |
| BCL2L2-PABPN1 | ECM1 | LHPP |
| IFNG | NEB | GPN1 |
| GNAI3 | ERLIN2 | MIR146A |
| PLCB4 | PLXND1 | MIR1269B |
| SPEG | ZNF462 | GZMB |
| ETV5 | VPS53 | IL5 |
| MSX1 | SRPX2 | EYA1 |
| NFKB1 | RIF1 | CDKN1A |
| IRF6 | ADGRG1 | SRC |
| SLC29A3 | TTC7A | SOD1 |
| DLG1 | SPATA22 | RHOA |
| NECTIN1 | TEX49 | CNTNAP2 |
| CR2 | DM1-AS | HCRT |
| CD19 | MBS1 | GGT1 |
| GRIN2B | LOC107075317 | GP6 |
| PI4KA | LOC109461477 | GP5 |
| ADNP | LOC109461479 | PLAUR |
| DMPK | PTLS | AKT1 |
| F12 | SOX3 | PTCH1 |
| CDKN2A | FASLG | TWIST1 |
| MYD88 | MIR126 | MSN |
| DGCR6L | MIR17 | CPA6 |
| MIR21 | MIR423 | PITX2 |
| IL4 | MIR338 | CASP9 |
| GSC2 | ICAM1 | MYH7 |
| H2AC18 | AIRE | NRG1 |
| HLA-A | TBX22 | CYP2C9 |
| FGF10 | LBR | MMP3 |
| MIR29C | TBX5 | MYOD1 |
| ASAH1 | SLC25A1 | XRCC3 |
| ARHGAP29 | CDC45 | CD46 |
| DLX4 | TBX2 | CD79A |
| BCL2 | DISC1 | TGM2 |
| HLA-B | RANBP1 | AP3B1 |
| DGCR | LZTR1 | MECP2 |
| HLA-DQB1 | TBX20 | IL13 |
| TAF1 | SNAP29 | NPPB |
| HTT | CLTCL1 | MT-CO1 |
| ALDH2 | SCARF2 | AQP1 |
| GSTM3 | ZDHHC8 | GRIN2A |
| EDA | ZNF74 | TH |
| HOXA3 | HIC2 | CREB1 |
| PTEN | SEPTIN5 | EPCAM |

Supplementary table 3. Top 10 GO enrichment of Biological Processes (BP), Cellular Components (CC) and Molecular Functions (MF) with their representative enriched terms (one per cluster). "Count" is the number of genes in the user-provided lists with membership in the given ontology term."Log10(P)" is the p-value in log base 10.

| **GO** | **Category** | **Description** | **Count** | **Log10(P)** |
| --- | --- | --- | --- | --- |
| GO:0010035 | GO Biological Processes | response to inorganic substance | 25 | -27.51 |
| GO:0042493 | GO Biological Processes | response to drug | 20 | -22.86 |
| GO:0032496 | GO Biological Processes | response to lipopolysaccharide | 18 | -20.86 |
| GO:0042110 | GO Biological Processes | T cell activation | 18 | -18.16 |
| GO:0030335 | GO Biological Processes | positive regulation of cell migration | 18 | -17.1 |
| GO:0072593 | GO Biological Processes | reactive oxygen species metabolic process | 14 | -16.78 |
| GO:0097190 | GO Biological Processes | apoptotic signaling pathway | 18 | -16.75 |
| GO:0071407 | GO Biological Processes | cellular response to organic cyclic compound | 18 | -16.71 |
| GO:0001934 | GO Biological Processes | positive regulation of protein phosphorylation | 19 | -16.32 |
| GO:0051384 | GO Biological Processes | response to glucocorticoid | 12 | -16.1 |
| GO:0045121 | GO Cellular Components | membrane raft | 10 | -9.1 |
| GO:0098552 | GO Cellular Components | side of membrane | 9 | -5.54 |
| GO:0031968 | GO Cellular Components | organelle outer membrane | 6 | -5.29 |
| GO:0048471 | GO Cellular Components | perinuclear region of cytoplasm | 9 | -4.99 |
| GO:0043235 | GO Cellular Components | receptor complex | 7 | -4.18 |
| GO:0000307 | GO Cellular Components | cyclin-dependent protein kinase holoenzyme complex | 3 | -4.17 |
| GO:0009898 | GO Cellular Components | cytoplasmic side of plasma membrane | 4 | -3.49 |
| GO:0098685 | GO Cellular Components | Schaffer collateral - CA1 synapse | 3 | -3.34 |
| GO:0031012 | GO Cellular Components | extracellular matrix | 6 | -3.12 |
| GO:0031965 | GO Cellular Components | nuclear membrane | 4 | -2.6 |
| GO:0005126 | GO Molecular Functions | cytokine receptor binding | 14 | -16.02 |
| GO:0019903 | GO Molecular Functions | protein phosphatase binding | 8 | -9.34 |
| GO:0019900 | GO Molecular Functions | kinase binding | 12 | -7.79 |
| GO:0032813 | GO Molecular Functions | tumor necrosis factor receptor superfamily binding | 5 | -7.42 |
| GO:0101021 | GO Molecular Functions | estrogen 2-hydroxylase activity | 3 | -7.18 |
| GO:0044389 | GO Molecular Functions | ubiquitin-like protein ligase binding | 8 | -6.82 |
| GO:0004935 | GO Molecular Functions | adrenergic receptor activity | 3 | -6.26 |
| GO:0042803 | GO Molecular Functions | protein homodimerization activity | 10 | -6.24 |
| GO:0002020 | GO Molecular Functions | protease binding | 5 | -5.21 |
| GO:0008134 | GO Molecular Functions | transcription factor binding | 8 | -4.84 |

Supplementary table 4 **Top 20 KEGG pathway**

| **Description** | **Count** | **pvalue** | **Target** |
| --- | --- | --- | --- |
| AGE-RAGE signaling pathway in diabetic complications | 15 | 2.49E-17 | AKT1/BCL2/TNF/CASP3/STAT1/ICAM1/SELE/VEGFA/CCND1/IL6/F3/IL1B/CXCL8/IL1A/STAT3 |
| Lipid and atherosclerosis | 18 | 4.1E-16 | AKT1/BCL2/TNF/CASP3/CYP1A1/ICAM1/SELE/PPP3CA/MMP3/CASP9/MMP9/IL6/TP53/CASP8/IL1B/CXCL8/CD40LG/STAT3 |
| Bladder cancer | 11 | 6.84E-16 | EGFR/VEGFA/CCND1/CDKN1A/MMP9/TP53/ERBB2/MYC/CXCL8/RASSF1/CDH1 |
| Kaposi sarcoma-associated herpesvirus infection | 17 | 1.49E-15 | PTGS2/AKT1/CASP3/STAT1/ICAM1/PPP3CA/VEGFA/CCND1/CDKN1A/CASP9/IL6/TP53/CASP8/HIF1A/MYC/CXCL8/STAT3 |
| Human cytomegalovirus infection | 17 | 1.8E-14 | PTGS2/AKT1/TNF/CASP3/PPP3CA/EGFR/VEGFA/CCND1/CDKN1A/CASP9/IL6/TP53/CASP8/MYC/IL1B/CXCL8/STAT3 |
| Hepatitis B | 15 | 4.08E-14 | CCNA2/AKT1/BCL2/TNF/CASP3/STAT1/CDKN1A/CASP9/MMP9/IL6/TP53/CASP8/MYC/CXCL8/STAT3 |
| Epstein-Barr virus infection | 15 | 1.08E-12 | CCNA2/AKT1/BCL2/TNF/CASP3/STAT1/ICAM1/CCND1/CDKN1A/CASP9/IL6/TP53/CASP8/MYC/STAT3 |
| Measles | 13 | 2.3E-12 | AKT1/BCL2/CASP3/STAT1/CCND1/CASP9/IL6/TP53/CASP8/IL1B/IL2/IL1A/STAT3 |
| Hepatitis C | 13 | 1.11E-11 | AKT1/TNF/CASP3/STAT1/EGFR/CCND1/CDKN1A/CASP9/TP53/CASP8/MYC/IFNG/STAT3 |
| Pancreatic cancer | 10 | 3.34E-11 | AKT1/STAT1/EGFR/VEGFA/CCND1/CDKN1A/CASP9/TP53/ERBB2/STAT3 |
| Chemical carcinogenesis - receptor activation | 14 | 3.43E-11 | PGR/ADRB2/ADRB1/AKT1/BCL2/CYP3A4/CYP1A2/CYP1A1/GSTM1/EGFR/VEGFA/CCND1/MYC/STAT3 |
| Fluid shear stress and atherosclerosis | 12 | 4.69E-11 | AKT1/BCL2/TNF/ICAM1/SELE/GSTM1/VEGFA/MMP9/TP53/IL1B/IFNG/IL1A |
| Endometrial cancer | 9 | 7.58E-11 | AKT1/EGFR/CCND1/CDKN1A/CASP9/TP53/ERBB2/MYC/CDH1 |
| TNF signaling pathway | 11 | 8.14E-11 | PTGS2/AKT1/TNF/CASP3/ICAM1/SELE/MMP3/MMP9/IL6/CASP8/IL1B |
| IL-17 signaling pathway | 10 | 2.91E-10 | PTGS2/TNF/CASP3/MMP3/MMP9/IL6/CASP8/IL1B/CXCL8/IFNG |
| Proteoglycans in cancer | 13 | 3.22E-10 | AKT1/TNF/CASP3/EGFR/VEGFA/CCND1/CDKN1A/MMP9/TP53/HIF1A/ERBB2/MYC/STAT3 |
| Prostate cancer | 10 | 3.98E-10 | AKT1/BCL2/MMP3/EGFR/CCND1/CDKN1A/CASP9/MMP9/TP53/ERBB2 |
| Non-small cell lung cancer | 9 | 5.66E-10 | AKT1/EGFR/CCND1/CDKN1A/CASP9/TP53/ERBB2/RASSF1/STAT3 |
| Influenza A | 12 | 5.71E-10 | AKT1/TNF/CASP3/STAT1/ICAM1/CASP9/IL6/CASP8/IL1B/CXCL8/IFNG/IL1A |
| Platinum drug resistance | 9 | 6.43E-10 | AKT1/BCL2/CASP3/GSTM1/CDKN1A/CASP9/TP53/CASP8/ERBB2 |
